# Osteogenic shift in the adipose-derived stem cells of *Acomys cahirinus* is linked to impaired adipose tissue self-renewal

**DOI:** 10.3389/fcell.2025.1603405

**Published:** 2025-07-30

**Authors:** M. Boldyreva, E. Zubkova, E. Trubkina, M. Agareva, S. Michurina, N. Alekseeva, I. Beloglazova, E. Ratner, Ye Parfyonova, I. Stafeev

**Affiliations:** ^1^ Angiogenesis Lab, Institute of Experimental Cardiology, Chazov National Medical Research Centre for Cardiology, Moscow, Russia; ^2^ Faculty of Biology and Biotechnology, National Research University “Higher School of Economics”, Moscow, Russia; ^3^ Faculty of Basic Medicine, Lomonosov Moscow State University, Moscow, Russia; ^4^ Faculty of Biology, Lomonosov Moscow State University, Moscow, Russia

**Keywords:** *Acomys cahirinus*, adipose-derived stem cells, adipogenesis, osteogenesis, regeneration

## Abstract

**Background:**

In recent years, there has been a significant increase in interest in *Acomys cahirinus* due to their unique regenerative properties and specific metabolism. We propose that *Acomys sp*. adipose-derived stem cells (ADSC) may have unique properties allowing them to adapt to caloric overload and prevent severe metabolic abnormalities. ADSC characterization from *Acomys cahirinus* may reveal novel pro-regenerative targets and provide insight into mechanisms enabling the maintenance of metabolic health during obesity.

**Methods:**

ADSCs were isolated from the subcutaneous fat depots of *Acomys cahirinus* and *Mus musculus,* which was used as a classic lab animal rodent model. The mesenchymal phenotype of ADSC was confirmed by surface markers expression and differentiation ability. Proliferation and migration of ADSC were assessed by metabolic tests and microscopy. Osteogenesis and adipogenesis were evaluated by specific staining and RT PCR gene expression analysis. Subcutaneous adipose tissue was characterized by histology and Western blotting.

**Results:**

*Acomys cahirinus* ADSC exhibited classic mesenchymal phenotype. Proliferation and wound healing were more active in *Acomys cahirinus* ADSC. These ADSC demonstrated enhanced osteogenesis and suppressed adipogenesis. *Acomys cahirinus* has larger adipose tissue depots than *Mus musculus* and lower blood glucose level. *Acomys cahirinus* adipose tissue is distinguished by lowered proliferation, enlarged adipocytes and suppressed adipose tissue triglyceride lipase (ATGL) expression.

**Conclusion:**

We conclude that *Acomys cahirinus* ADSC have high regenerative potential. Nevertheless, the augmented osteogenic capacity of *Acomys cahirinus* ADSC can be related with a limited ADSC participation in adipose tissue self-renewal. The reduction in ATGL expression observed in *Acomys cahirinus* adipose tissue may serve as a protective mechanism in the face of excessive adipose tissue accumulation.

## 1 Introduction

Over the past 2 decades, there has been a notable surge in interest in spiny mice (*Acomys* species) as a subject of scientific inquiry due to their remarkable regenerative capacity and obese phenotype (Gaire et al., 2021; Sandoval and Maden, 2020; Shafrir et al., 2006). Regeneration in *Acomys cahirinus* has been extensively characterized in the context of skin regrowth (Seifert et al., 2012; Jiang et al., 2019; Brant et al., 2016), restoration of ear tissues (Matias Santos et al., 2016; Gawriluk et al., 2016), skeletal muscles (Maden et al., 2018), spinal cord and heart (Nogueira-Rodrigues et al., 2022; Streeter et al., 2019; Qi et al., 2021) after severe injuries. However, increased regeneration can have beneficial effects not only on wound healing, but also on self-renewal of tissues and organs supporting their plasticity. Adipose tissue renewal represents a crucial aspect of maintaining metabolic homeostasis during obesity (Vorotnikov et al., 2019; Monji et al., 2022). As long as *Acomys sp*. are known to exhibit obesity with normoglycemia (Shafrir et al., 2006; Shafrir, 2000), we propose that this phenomenon can be attributed to the distinctive characteristics of adipose tissue progenitor cells.

In their natural habitat, *Acomys cahirinus* are known to subsist on a diet comprising a high proportion of fiber and protein. In laboratory conditions with *ad libitum* feeding of a chow diet supplemented with fatty seeds, *Acomys cahirinus* are susceptible to developing obesity due to a tendency to overeat (Haughton et al., 2016). However, elevated fat consumption and the obese phenotype in *Acomys cahirinus* are not associated with pronounced hyperglycemia. While blood glucose level had been reported to increase on fat-rich diet in spiny mice (Shafrir et al., 2006), in comparison to mouse strains C57BL/6J and Swiss albino, *Acomys cahirinus* exhibit lower blood glucose level and downregulated insulin release in response to elevated blood glucose (Gutzeit et al., 1979). Apparently, in *Acomys cahirinus* glucose is transported predominantly via insulin-independent mechanisms into such glucose-utilizing tissues as skeletal muscle and fat, accounting for low basal blood glucose concentration (Gonet et al., 1966). We therefore propose that adipose tissue plasticity and self-renewal may play a pivotal role in maintaining the non-diabetic phenotype in obese *Acomys cahirinus* fed with a seed-rich diet.

The expansion of adipose tissue in obesity can be attributed to either cell hypertrophy or hyperplasia. The formation of new adipocytes is advantageous over the enlargement of existing adipocytes, which is closely related to metabolic abnormalities (Lemoine et al., 2012; Kawai et al., 2021). Adipose-derived stem cells (ADSC) are the primary source of preadipocytes, and their proliferative and differentiation potential are crucial for adipose tissue renewal and optimal functionality. Indeed, downregulated proliferation and shift of differentiation from adipo-to osteogenesis in ADSC associates to the type 2 diabetes development among obese individuals (Stafeev et al., 2019; Skubis-Sikora et al., 2020; Agareva et al., 2022). We suggest that *Acomys cahirinus* ADSC may have unique properties that enable them to adapt to caloric overload and prevent severe metabolic abnormalities.

In addition to their role in adipose tissue health, ADSC are also emerging as a prominent cell therapy option in regenerative medicine. Native ADSC are widely used for peripheral artery disease therapy, heart regeneration, treatment of diabetes and other pathologies (Silvestre et al., 2016; Gautam et al., 2015; Liao et al., 2017). Secretome of ADSC has high translational potential to clinical practice and has already been applied for spermatogenesis activation, neurodegenerative disease therapy, wound healing, etc (Sagaradze et al., 2019; Ghasemi et al., 2023; Alaniz et al., 2023). Moreover, genetic modification of ADSC may improve their properties and facilitate their utilization for bone, cardiovascular and other pathologies (Lien et al., 2009; Boldyreva et al., 2019). In summary, the characterization of ADSC from *Acomys cahirinus* may reveal novel pro-regenerative targets and provide insight into mechanisms enabling the maintenance of metabolic health during obesity. As a reference of classic small animal rodent model we have used *Mus musculus*–the most useful small lab animal in huge number of studies.

## 2 Materials and methods

### 2.1 Animal housing and characterization

Healthy male *Mus musculus* C57BL/6J mice (weight 20–22 g) were purchased from “Andreevka” animal husbandry facility, Russia. Healthy male *Acomys cahirinus* (weight 33–34 g) were obtained from our in-house breeding colony. Healthy male *Rattus norvegicus* (weight 120–140 g) for flow cytometry experiment were purchased from “Andreevka” animal husbandry facility. Male animals were used according to the best practice in metabolic studies: male animals have less variability for the reason of menstruation cycle absence. Both animals were selected randomly, for experiments we have used 10 animals per group. All selected animals were maintained in ventilated cages at 25 ± 2°C temperature, with a light/dark cycle of 12h/12h per day under standard pathogen-free conditions with sterile chow and water *ad libitum*. Diet for C57BL/6J was standard diet for lab rodents (#PK120, Gatchina Animal Complete Feeds Factory, Russia). Diet for Acomys sp. was diet for decorative rodents (#855582066, LittleShark, Russia) with supplementation by dried *Hermetia illucens* imagoes (3 per week for one animal). Animal experiments were performed in accordance with the internal “Rules for conducting work using experimental animals”, ARRIVE guideline and were approved by the Ethical Board of the Chazov National Medical Research Center of Cardiology (permit # 3/24, 03.04.2024). Obtained results were used completely without any exclusion. Body weight of animals was measured at standard lab weigh-scale. Lee index, a BMI value for animals, was calculated as three squareroot body weight (g)/nasoanal length (cm)]*1000. Blood glucose level was measured by a Contour Plus One glucometer (Ascensia Diabetes Care, Basel, Switzerland).

### 2.2 ADSC isolation and subculturing

For tissue harvesting animals were euthanized through 5% isoflurane inhalation followed by cervical dislocation. Subcutaneous inguinal fat depots were isolated from animals in aseptic conditions and weighted at standard lab weigh-scale. Fat samples (300–600 mg) were dissected by surgical scissors on small pieces 1–2 mm^3^ in DMEM low glucose (DMEM LG, 1 g/L, Gibco, United States) supplemented by PenStrep (Servicebio, China) with consequent addition of collagenase I (200 U/mL, Worthington, United Kingdom) and dispase (30 U/mL, Gibco, United States) solutions and thorough resuspension, enzyme solutions volume was 5 mL. Tissue homogenate was incubated at 37°C for 30–45 min up to perlaceous tone with constant shaking, after that cell suspension was resuspended thoroughly and centrifuged at 350g for 10 min. The pellet was resuspended directly in DMEM LG + 10% FBS without passing through a cell strainer for the combination of pure cells and small explants. The suspension was seeded into culture plates and incubated at 37°C in 5% CO2 for expansion. ADSC were subcultures at 70%–80% confluence using Versen and trypsin solutions.

### 2.3 Flow cytometry

For *Acomys cahirinus* ADSC characterization, the expression of surface markers was analyzed by flow cytometry using antibodies specific to CD73 (ab175396, Abcam, United States), CD90 (PA5-80127, ThermoFischer, United States) and CD140b (ab32570 Abcam, United States). ADSC from *Acomys cahirinus*, *Mus musculus* C57BL/6J or *Rattus norvegicus* Wistar at passages 2-3 were fixed in 2% paraformaldehyde (PFA, Panreac, United States). Fixed cells were blocked in PBS with 1% bovine serum albumin (BSA, Sigma-Aldrich) and cells were then incubated with the primary antibodies or relevant IgG controls. Cells were subsequently incubated with the respective fluorophore-conjugated secondary antibody (Alexa Fluor 488 or Alexa Fluor 647, Invitrogen, United States) in the dark. Flow cytometry analysis was performed using a BD FACS Canto II cytometer (BD Biosciences) with BD FACSDiva software. Approximately 10,000 events per sample were recorded. Data were analyzed using Flowing software (v2.5.1 Turku University.).

### 2.4 ADSC differentiation potential analysis

Besides expression of specific surface markers, ADSC should have potential for three differentiation types (adipogenic, osteogenic, chondrogenic) according to The International Society for Cellular Therapy statement (Dominici et al., 2006). We have analyzed all three differentiation types according to protocols described below.

#### 2.4.1 Adipogenic differentiation

The adipogenic differentiation of ADSC was carried out according to the following protocol. Cells were cultured until reaching 100% confluency, after which the culture medium was replaced with an adipogenic induction medium (DMEM LG supplemented with 10% FBS, 1% PenStrep, 1 μg/mL insulin, 0.5 mM IBMX, 1 µM dexamethasone, 2 µM rosiglitazone) or a control medium (DMEM LG with 10% FBS and 1% PenStrep). Cells were maintained at 37°C in a humidified atmosphere with 5% CO2, the medium was changed every 3 days. The differentiation process lasted 21 days, after which the cells were used for further experiments. Visualization performed by lipophilic dye BODIPY493/503 staining (neutral lipids stain by green colour in fluorescence mode).

#### 2.4.2 Osteogenic differentiation

The osteogenic differentiation of ADSC was carried out according to the following protocol. Cells were cultured until reaching approximately 60% confluency, after which the culture medium was replaced with an osteogenic induction medium (DMEM LG supplemented with 10% FBS, 1% PenStrep, 100 nM dexamethasone, 10 mM sodium-β-glycerophosphate, and 50 uM sodium ascorbate) or a control medium (DMEM LG with 10% FBS and 1% PenStrep). Cells were maintained at 37°C in a humidified atmosphere with 5% CO2, the medium was changed every 3 days. The differentiation process lasted 28 days, after which the cells were used for further experiments. Visualization performed by Alizarin Red S staining: cells were fixed in 4% paraformaldehyde and stained by Alizarin Red S solution during 1 h with consequent washing by distilled water and visualization in Zeiss Axio Observer A1 microscope.

#### 2.4.3 Chondrogenic differentiation

The chondrogenic differentiation of ADSC was performed as follows. Initially, cells were cultured in DMEM LG supplemented with 10% FBS and 1% PenStrep. To establish a 3D environment, 150 µL of 2% agarose was added to wells of a 48-well plate, forming a supportive matrix. Next, 200 µL of cell suspension at a density of 2 million cells/mL was layered onto the agarose. After a two-day incubation, the culture medium was replaced with a chondrogenic induction medium (DMEM LG with 10% FBS, 1% PenStrep, 100 nM dexamethasone, 1% sodium pyruvate, 10 ng/mL TGF-β1, 1 μg/mL insulin). Cells were maintained at 37°C in a humidified atmosphere with 5% CO_2_, with medium changes every 3 days. After 21 days, the resulting cell spheroids were frozen in Tissue-Tek. Visualization performed by Alcian Blue staining: spheroids were fixed in 4% paraformaldehyde and stained by Alcian Blue solution during 1 h with consequent washing by distilled water and visualization in Zeiss Axio Observer A1 microscope.

### 2.5 Proliferation analysis

Proliferation has been analyzed by colorimetric and immunochemical methods. As a colorimetric method we have used MTT assay. ADSC at 3rd passage were seeded into 96-well plates in concentration of 4000 cells/well. Cells were deprived in DMEM LG with 1% FBS for the silence of all hormonal signals and cell cycle alignment and then stimulated with 10% FBS during 24 h. Nextly, we added 0.2 volume of MTT reagent solution (5 mg/mL) in ADSC culture medium to each well (1 volume) and incubated for 3 h at 37°C. Then the medium was fully removed from each well, and purple formazan insoluble crystals were dissolved in isopropanol for 5 min in the dark with constant stirring at room temperature. Absorbance for each well was measured at wavelength 490 nm on a VictorX3 plate spectrophotometer (Perkin-Elmer, United States). Immunochemical method included analysis of PCNA expression in cell nuclei. ADSC at 3rd passage were seeded into 6-well plates with coverslips (Corning, United States). Serum treatment scheme was similar to the MTT assay. After that, cells were fixed in cold 100% methanol for 5 min and were permeabilized with 0.2% Triton X-100 solution. Then samples were blocked in PBS with 10% normal goat serum for 45 min at room temperature. After that, the coverslips were boiled in antigen unmasking solution (Vector Laboratories, United States) and incubated with PCNA primary antibodies (ab29, Abcam, United States) overnight at 4°C. Secondary anti-mouse antibodies conjugated with AlexaFluor488 (ab150113, Abcam, United States) were used for detection. Samples were mounted in VECTASHIELD^®^ Antifade Mounting Medium with DAPI (Vector Laboratories, United States). Samples visualization was performed on a confocal microscope Leica Stellaris 5 (Leica Microsystems, Germany). For each group we obtained 15 fields of view (FOV) for further quantitative analysis in FIJI software. Obtained results allow evaluating ADSC ability to cytokines-stimulated proliferation.

### 2.6 Migration analysis

For the migration analysis we have used wound scratch assay which allows us to estimate cell migration and proliferation. ADSC at 2nd passage isolated from *Acomys cahirinus* and *Mus musculus* C57BL/6J were seeded into 12-well plates and grown to a full confluent monolayer. To synchronize the cells and minimize proliferation effects, the monolayers were incubated overnight in serum-free DMEM LG before performing the scratch assay. The scratch migration assay was performed by creating a wound in the cell monolayer using a sterile 200 µL yellow pipette tip (“Corning”, United States). The wells were then washed three times with phosphate-buffered saline (PBS) to remove detached cells and debris, leaving a clean wound edge. Images of the scratch area were captured at the start point (immediately after the scratch, 0 h), 6, 12, and 24 h. The total scratch area at each time point was quantified using ImageJ software (NIH, United States). The wound area was calculated as the remaining open area of the scratch at each time point, and wound closure was expressed as a percentage of the initial area (0 h). The following formula was used to calculate wound closure:

$$\text{Wound }\text{Closure }\left(\%\right)=\left(\text{Initial}\;\text{Area}‐\text{Area}\;\text{at}\;\text{Time}\;\text{Point}\;\text{Initial}\;\text{Area}\right)\times 100$$

### 2.7 Lipid droplets analysis

Following adipogenic differentiation, adipocytes were stained with 0.25 μg/mL BODIPY493/503 (Invitrogen, United States) for 20 min. After that cells were washed three times with DPBS and visualized in DMEM without phenol red. Visualization of BODIPY493/503 staining was performed using a confocal microscope Leica Stellaris 5 (Leica, Germany) in a CO2 and temperature controlled chamber. Three cellular replicates were performed, each of which analyzed 5 fields of view. Size and number of lipid droplets were analyzed in NIS-Elements software.

### 2.8 Real-time PCR

Total RNA was isolated from cells using the CleanRNA Standard kit (Evrogen, Russia). RNA concentration and purity were determined using the Nanodrop 2000 micro spectrometer (Thermo Scientific, United States). The isolated total RNA and the RevertAid H Minus First Strand cDNA kit (Thermo Scientific, United States) were used to synthesize cDNA. Each reaction mixture contained 1 μg of total RNA, so that the amount of synthesized cDNA in the reaction tubes was considered to be the same. A SYBR-green based gene expression assay was performed by RT PCR kit (Sintol, Russia). Primers for RT PCR calculated in PrimerBLAST, RNA sequences for *Acomys cahirinus* were used from SequenceServer database (Dickinson et al., 2012). Primers were synthesized in Evrogen, Russia (Supplementary Table S1). All samples were assayed in triplicates and values were compared against the housekeeping gene beta-actin. The mRNA level was quantified by the 2^(DDCt) method.

### 2.9 Western blotting

For protein analysis cells were lysed in RIPA buffer. Cell extracts were separated by Laemmli SDS-PAGE. Proteins were transferred from gel to PVDF membrane; they were blocked by 5% of fat-free milk solution on TBST and incubated with primary and secondary antibodies according to manufacturer’s instructions. Primary antibodies: anti-IRS-1 (#3407, Cell Signaling, United States); anti-FABP4 (#3544, Cell Signaling, United States); anti-ATGL (A5126, Abclonal, China); anti-ATP5A antibody or Complex V (#ab14748, Abcam, United States), anti-b-actin (AC026, Abclonal, China); secondary antibodies: HRP-conjugated goat anti-rabbit antibody (#AS014, Abclonal, China); HRP-conjugated goat anti-mouse antibody (#AS003, Abclonal, China). Stained protein bands were visualized using Clarity ECL kit (BioRad, United States) and Fusion FX gel-documentation system (Vilber Lourmat, France) in the video mode to ensure that digital results were in the linear range. Optical density quantification was performed using the GelAnalyzer 19.1 software (www.gelanalyzer.com, accessed on 1 July 2021; software by Istvan Lazar Jr., PhD and Istvan Lazar Sr., PhD, CSc; Budapest, Hungary).

### 2.10 Hematoxylin/eosin staining of adipose tissue samples

Adipose tissue samples from inguinal, epididymal and brown fat depots were isolated surgically under control of a surgical microscope. Samples were fixed in 10% PFA for 24 h. Nextly, adipose tissue samples were embedded in paraffin and 2 um thick sections were cut from the paraffin blocks. For staining, the selected sections were deparaffinized in xylene, and hydrated with graded ethanol. Staining by Mayer’s hematoxylin solution was performed for 2 min and dye was differentiated in flowing water (1 min). After that the slides were stained in eosin B solution (3 min). The slides were washed with 70% ethanol and incubated subsequently with graded ethanol and xylene. Slides were mounted in Cytoseal-60 (Richard-Allen Scientific, United States) under coverslips. Staining was visualized on scanning microscope Leica ScanScope CS (Leica Microsystems, Germany) and analyzed by Aperio ImageScope software. Adipocytes average size was determined using ImageExFluorer software.

### 2.11 Immunohistochemical staining of adipose tissue samples

Samples were prepared as described above, and sections were incubated in a citrate buffer for antigen retrieval (10 mM citric acid, pH 6.0) for 45 min at 60°C to unmask the antigen. For classic IHC, 3% hydrogen peroxide was added for 10 min. Sections were blocked with 10% bovine serum albumin (BSA) for 30 min at 21°C and incubated with primary antibodies anti-PCNA (ab29, Abcam). Subsequently, sections were incubated with horseradish peroxidase-conjugated secondary antibodies for 1 h at room temperature. Finally, 3, 3-diaminobenzidine was used to observe the chromogen, and hematoxylin was used for counterstaining. Staining was visualized on scanning microscope Leica ScanScope CS (“Leica Microsystems”, Germany) and analyzed by Aperio ImageScope software. For the analysis, 5 fields of view were selected from each representative slice. Quantitative analysis was performed in FIJI software (United States). For immunofluorescence IHC sections were blocked with 10% normal goat serum for 1 h at room temperature and incubated with primary antibodies anti-ATGL (A5126, Abclonal, China). Subsequently, sections were incubated with Alexa488-conjugated secondary antibodies for 1 h at room temperature. Finally, DAPI was used for counterstaining. Staining was visualized on confocal microscope Leica Stellaris 5 (“Leica Microsystems”, Germany) and analyzed by LasX software. For the analysis, 5 fields of view were selected from each representative slice. Quantitative analysis was performed in FIJI software (United States).

### 2.12 Statistical analysis

Graphs were generated in GraphPad Prism 8.0 and data are presented as mean ± standard error mean (SEM) for each group. Mann-Whitney rank sum U-test was employed to evaluate the difference between two independent groups. For multiple comparisons we used Kruskell-Wallis test with post hoc Dunn’s test. The threshold for statistical significance was set at p < 0.05.

## 3 Results

### 3.1 ADSC isolated from *Acomys cahirinus* exhibit mesenchymal phenotype

The main goal of our study is to elucidate the characteristics of ADSC from the subcutaneous adipose depot of *Acomys cahirinus*. Subcutaneous fat depot is a first tissue which meet caloric overload and participate in the obesity development. The initial phase of our investigation entailed substantiating the mesenchymal phenotype of the isolated cells in accordance with the International Society for Cellular Therapy statement, which had not previously been done. In flow cytometry experiments we have used cells of *Mus musculus* (C57BL/6J strain) and *Rattus norvegicus* (Wistar strain) as a control. Using of two reference animals caused by differences between protein sequences and conformations of classic lab rodents and *Acomys cahirinus*. Moreover, flow cytometry has the highest requirements for native proteins conformation and some *Acomys cahirinus* proteins can not be detected by appropriate for *Mus musculus* proteins detection antibodies. In this case, we are forced to use *Rattus norvegicus* protein detection antibodies and respective cell control.

**FIGURE 1:**
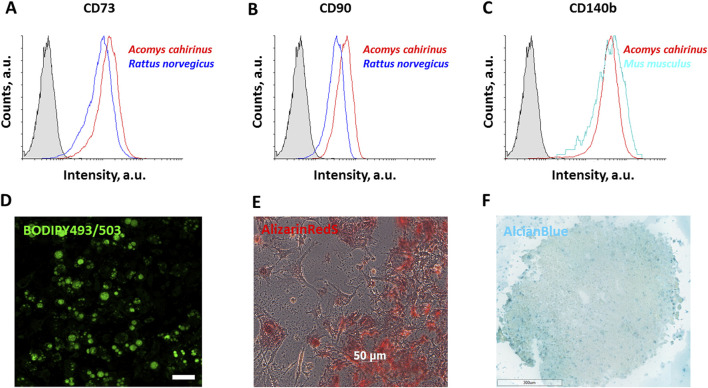
ADSC isolated from *Acomys cahirinus* display mesenchymal surface markers and the capacity for multilineage differentiation. **(A-C)** - flow cytometry results of surface markers expression: CD73 **(A)**, CD90 **(B)** and CD140b **(C)** in ADSC, 15,000 cells were detected for the complete analysis; **(D)** adipogenic differentiation of *Acomys cahirinus* ADSC, BODIPY493/503 staining; **(E)** osteogenic differentiation of *Acomys cahirinus* ADSC, AlizarinRedS staining; **(F)** chondrogenic differentiation of *Acomys cahirinus* ADSC, AlcianBlue staining. All experiments have been performed with n = 3.

To estimate the mesenchymal phenotype of *Acomys cahirinus* ADSC we examined the expression of specific surface markers. The results demonstrated that *Acomys cahirinus* ADSC exhibited equivalent expression of mesenchymal markers CD73, CD90, and CD140b as well-characterized ADSC from *Mus musculus* (Figures 1A–C). Next, we confirmed multiple differentiation potentials of *Acomys cahirinus* ADSC, which were able to accumulate lipids calcium inclusions and acidic glycosaminoglycans under adipogenic, osteogenic and chondrogenic stimuli, respectively (Figures 1D–F). Thus, ADSC from subcutaneous adipose tissue of *Acomys cahirinus* have mesenchymal phenotype and can be further compared with murine ADSC.

### 3.2 ADSC from *Acomys cahirinus* demonstrate enhanced proliferation and similar migratory activity in comparison to *Mus musculus* ADSC

As stated above in the Introduction subsection, *Acomys cahirinus* has unique regenerative capabilities. Consequently, our investigation focused on assessing *Acomys cahirinus* ADSC properties that could contribute to elevated regenerative potential, including proliferative and migratory activities of these cells. In all subsequent experiments, cells and tissue samples of *Mus musculus*, C57BL/6J strain, were used as a control.

**FIGURE 2:**
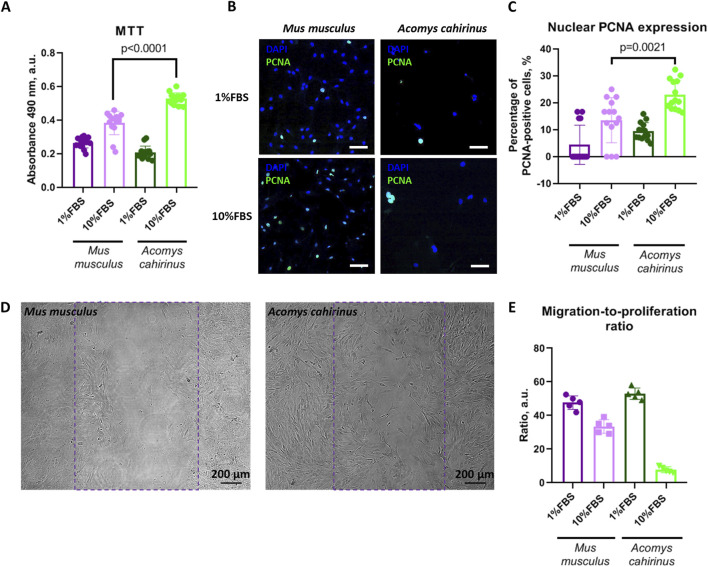
*Acomys cahirinus* ADSC have enhanced proliferative potential with equal migratory activity in comparison with *Mus musculus* ADSC. **(A)** analysis of ADSC proliferative activity by MTT test; **(B)** representative images of PCNA (proliferating cell nuclear antigen) staining in stimulated and non-stimulated ADSC, scalebar = 100 um; **(C)** quantification of PCNA-expressing cells in ADSC cultures; **(D)** representative images of migration analysis “scratch assay”, violet line marks initial scratch zone, scalebar = 200 um; **(E)** migration-to-proliferation ratio. The data are presented as mean ± SD; Mann-Whitney rank sum U-test was used to calculate significance of differences; significance threshold was set at p < 0.05. All experiments have been performed with cells from 5 animals per group.

In order to obtain an initial estimation, we employed the MTT test, which represents the most commonly utilized method for proliferation analysis based on cell metabolic activity. The *Acomys cahirinus* ADSC demonstrated markedly elevated proliferative activity in response to FBS (Figure 2A) when compared to the classic *Mus musculus* ADSC model. Nevertheless, the MTT results in the context of proliferation should be confirmed by alternative methods. To this end, we quantified the number of proliferating cells by immunocytochemistry of PCNA (proliferating cell nuclear antigen) in ADSC nuclei. The MTT data was confirmed by the results of PCNA expression analysis. Thus, *Acomys cahirinus* ADSC demonstrated enhanced proliferative capacity in comparison with the classic *Mus musculus* ADSC model (Figures 2B,C).

Migration is also a significant biological process that facilitates regeneration. We estimated migratory activity of ADSC by “wound scratch assay” - *in vitro* test on wound healing. The *Acomys cahirinus* ADSC exhibited a markedly elevated rate of wound healing in the presence of FBS (Figure 2D). However, the normalization of migration analysis results on proliferative activity indicated that the predominant mechanism underlying the accelerated wound healing was not migration enhancement but activated proliferation (Figure 2E). In conclusion, the *Acomys cahirinus* ADSC demonstrated enhanced regenerative potential, which was manifested by augmented proliferation in comparison with the classic model *Mus musculus* ADSC.

### 3.3 ADSC isolated from *Acomys cahirinus* have enhanced osteogenic activity

Not only proliferation of ADSC is important for adipose tissue renewal and maintaining metabolic health. Their ability to give rise to new adipocytes and maintain the balance between differentiation potentials is also essential.

**FIGURE 3:**
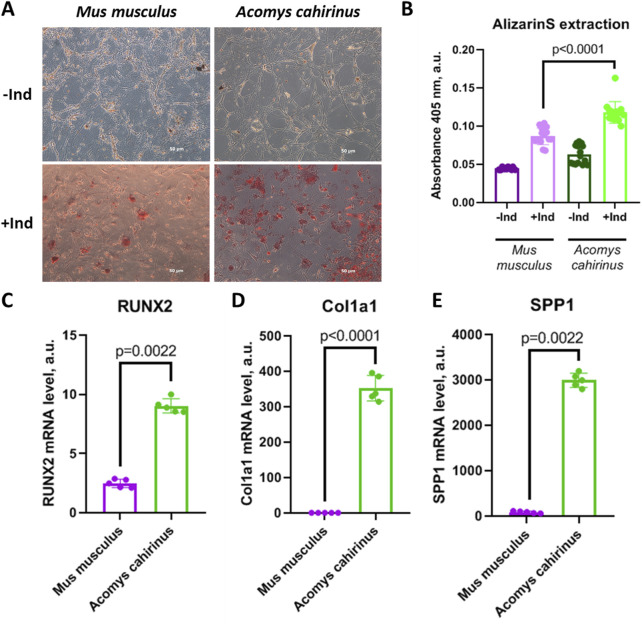
*Acomys cahirinus* ADSC exhibit enhanced osteogenesis in comparison with *Mus musculus* ADSC. **(A)** representative images of calcium inclusions stained with AlizarinRedS at 28 days of osteogenic differentiation, **(B)** quantification of AlizarinRedS accumulation by isopropanol extraction; **(C-E)** − mRNA levels of osteogenic markers RUNX2 **(C)**, Col1a1 **(D)** and SPP1 **(E)** at 28 days of osteogenic differentiation. The data are presented as mean ± SD; Mann-Whitney rank sum U-test was used to calculate significance of differences; significance threshold was set at p < 0.05. All experiments have been performed with cells from 5 animals per group.

For the analysis of *Acomys cahirinus* differentiation potential we have accurately analyzed osteogenesis from *Acomys cahirinus* ADSC in comparison with *Mus musculus* ADSC. We have shown that *Acomys cahirinus* ADSC have higher osteogenic potential than *Mus musculus* ADSC. The amount of calcium inclusions is markedly elevated in *Acomys cahirinus* ADSC, as evidenced by microscopy and AlizarinRedS extraction (Figures 3A,B). It should be noted that calcium inclusions are not a singular feature of osteogenesis. In addition, the expression of osteogenic markers was also analyzed. RUNX2 (Runt-related transcription factor 2) is the master regulator of osteogenesis, and its expression was observed to be 4-fold higher in *Acomys cahirinus* osteocytes (Figure 3C). The effector proteins collagen I and osteopontin (encoded by the Col1a1 and SPP1 genes) exhibited 400-fold and 40-fold higher expression, respectively, in *Acomys cahirinus* osteocytes (Figures 3D,E). It is evident that *Acomys cahirinus* ADSC exert greater osteogenic potential in comparison to *Mus musculus* ADSC.

#### 3.3.1 ADSC isolated from *Acomys cahirinus* have suppressed adipogenic activity

It is well established that the osteogenic and adipogenic differentiation programs in ADSC are in competition with one another. Consequently, upregulated osteogenesis can be associated with adipogenesis downregulation in *Acomys cahirinus* ADSC. In the next step of our study we have analyzed adipogenic differentiation of *Acomys cahirinus* ADSC in comparison with classic model *Mus musculus* ADSC.

**FIGURE 4:**
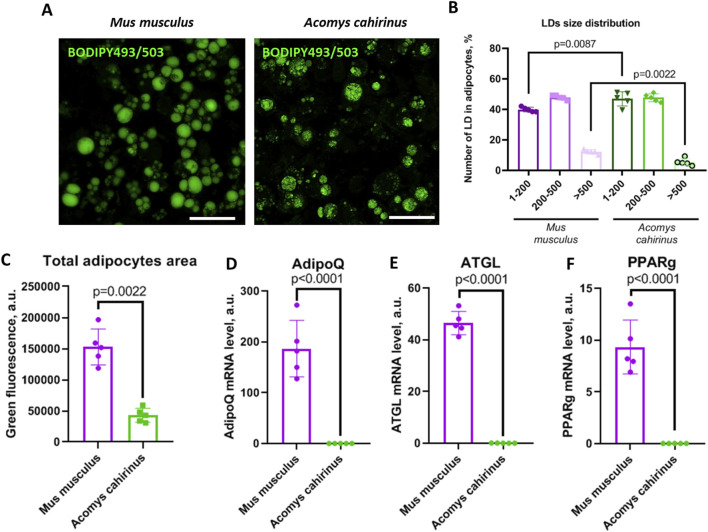
*Acomys cahirinus* ADSC exhibit suppressed adipogenesis in comparison with *Mus musculus* ADSC. **(A)** representative images of lipid droplets (LDs) stained with BODIPY493/503 at 21 days of adipogenic differentiation, **(B)** LDs size distribution in mature adipocytes at 21 days of adipogenic differentiation; **(C)** total adipocytes area in mature adipocytes at 21 days of adipogenic differentiation; **(D-F)** - mRNA levels of adipogenic markers AdipoQ **(D)**, ATGL **(E)** and PPARγ **(F)** at 21 days of adipogenic differentiation. The data are presented as mean ± SD; Mann-Whitney rank sum U-test was used to calculate significance of differences; significance threshold was set at p < 0.05. Scalebar = 150 um. All experiments have been performed with cells from 5 animals per group.

We observed that *Acomys cahirinus* ADSC exhibited suppressed adipogenic activity when compared with the classic *Mus musculus* ADSC model. The number of LDs in the *Acomys cahirinus* ADSC culture exposed to adipogenic inducers was found to be significantly lower than in control *Mus musculus* ADSC. (Figure 4A). Moreover, LDs size distribution analysis revealed an increase in the number of small LDs and a decrease in the number of large LDs in *Acomys cahirinus* adipocytes (Figure 4B). Also, it should be noted that *Acomys cahirinus* adipocytes had modified LDs morphology with a rugged surface, which allowed to hypothesize changes in the composition of LDs-associated proteins between *Acomys cahirinus* and *Mus musculus* (Figures 4A,C). *Acomys cahirinus* adipocytes displayed 6000-fold and 1000-fold lower mRNA expression levels of effector adipogenic markers adiponectin (AdipoQ) and adipocyte triglyceride lipase (ATGL), respectively, than *Mus musculus* ADSC (Figures 4D,E). Moreover, adipogenesis master regulator PPARγ also was significantly downregulated in *Acomys cahirinus* adipocytes (Figure 4F). Therefore, *Acomys cahirinus* ADSC demonstrated diminished adipogenic activity in comparison with classic model *Mus musculus* ADSC.

### 3.4 *Acomys cahirinus* has higher fat content and lower blood glucose level than *Mus musculus*

As long as downregulated differentiation ability of adipose progenitors may affect fat expansion and systemic metabolism, we decided to characterize subcutaneous adipose tissue depot and blood glucose level in *Acomys cahirinus*.

**FIGURE 5:**
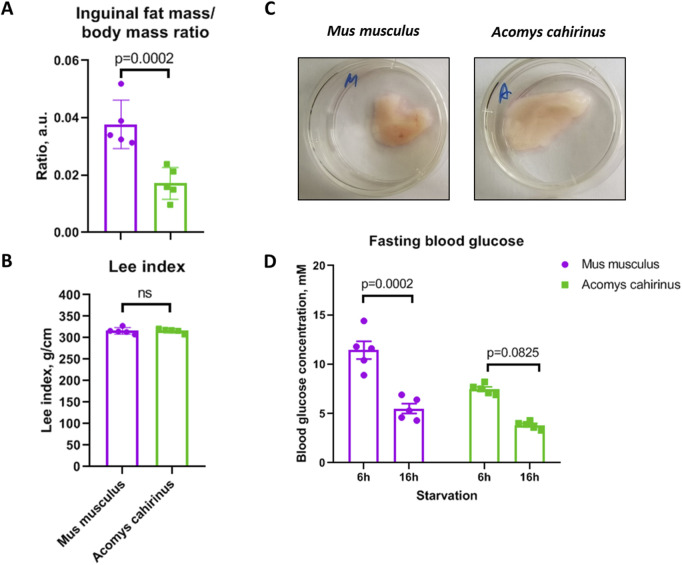
*Acomys cahirinus* exhibits elevated subcutaneous fat storage and decreased fasting blood glucose level in comparison to *Mus musculus*. **(A)** relative inguinal adipose tissue content normalized to body mass; **(B)** Lee obesity index in *Acomys cahirinus* and *Mus musculus*. **(C)** representative images of whole mount inguinal adipose tissue depots; **(D)** blood glucose level after 6h and 16h of fasting. The data are presented as mean ± SD; Mann-Whitney rank sum U-test was used to calculate significance of differences; significance threshold was set at p < 0.05. All experiments have been performed with cells from 5 animals per group.

We confirmed that the amount of subcutaneous adipose tissue is significantly elevated in *Acomys cahirinus* when compared to *Mus musculus,* while Lee obesity index, which correlates with body composition, is equal among these species (Figures 5A–C). At the same time, blood glucose level is lower in *Acomys cahirinus* than in *Mus musculus* and is 7.5 mM and 3.8 mM after 6 h and 16 h of fasting, respectively (Figure 5D). The data illustrate the distinctive metabolic characteristics of *Acomys cahirinus*, enabling these animals to sustain a state of obesity while maintaining normal glucose levels.

### 3.5 Adipose tissue of *Acomys cahirinus* is composed of enlarged adipocytes with decreased ATGL expression

According to our hypothesis, increased proliferation and downregulated adipogenesis of ADSC in *Acomys cahirinus* may have an influence on the adipose tissue renewal and metabolic state. To further estimate how ADSC properties may contribute to adipose tissue homeostasis *in vivo*, we compared adipocyte size, the number of proliferating cells and the expression of adipocyte triglyceride lipase as a marker of lipolytic activity in subcutaneous adipose tissues of *Acomys cahirinus* and *Mus musculus*.

**FIGURE 6:**
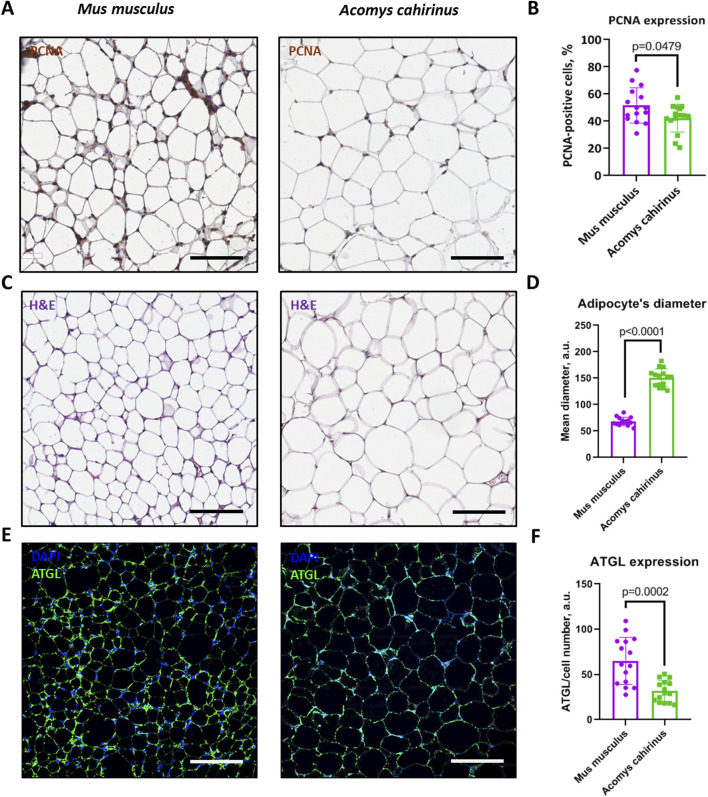
Adipose tissue of *Acomys cahirinus* contains a lower number of proliferating cells, larger adipocytes with decreased expression of adipose triglyceride lipase (ATGL). **(A)** representative images of PCNA (proliferating cell nuclear antigen) staining in subcutaneous adipose tissue of *Acomys cahirinus* and *Mus musculus*, **(B)** quantification of PCNA-positive cells in adipose tissue; **(C)** representative images of hematoxylin-eosin staining in subcutaneous adipose tissue of *Acomys cahirinus* and *Mus musculus;* **(D)** quantification adipocyte size in adipose tissue; **(E)** representative images of ATGL staining in subcutaneous adipose tissue of *Acomys cahirinus* and *Mus musculus*, **(F)** quantification of ATGL expression in adipose tissue. The data are presented as mean ± SD; Mann-Whitney rank sum U-test was used to calculate significance of differences; significance threshold was set at p < 0.05. Scale bar = 100 um. All experiments have been performed with cells from 5 animals per group.

We found that the number of proliferating cells with high PCNA expression was lower in *Acomys cahirinus* subcutaneous adipose tissue in comparison to *Mus musculus* (Figures 6A,B)*,* which contradicts the reported enhanced ADSC proliferation in *Acomys cahirinus* (Figures 2A–C). This inconsistency can be attributed to the detection of additional proliferating cell types within the adipose tissue in *Mus musculus*, such as immune cells. Next, we observed that *Acomys cahirinus* has enlarged adipocytes (Figures 6C,D) and significantly lower expression of lipolytic marker ATGL (Figures 6E,F). Our findings suggest that there may be an impairment of the adipocyte renewal in *Acomys cahirinus*, which appears to be compensated for by elevated fat accumulation and reduced triglyceride hydrolysis in existing mature adipocytes.

### 3.6 Expression of metabolic proteins in adipose tissue and ADSC-derived adipocytes of *Acomys cahirinus* and *Mus musculus*

In addition to excessive fat accumulation, other lipid handling and bioenergetic processes in adipose tissue may correspond to unique systemic metabolism of *Acomys cahirinus.* To provide an insight into how *Acomys cahirinus* is able to store enormous amount of fat and maintain low blood glucose level, we measured the expression of some proteins important for insulin signaling, lipid handling and bioenergetics in both subcutaneous adipose tissue and ADSC-derived adipocytes.

**FIGURE 7:**
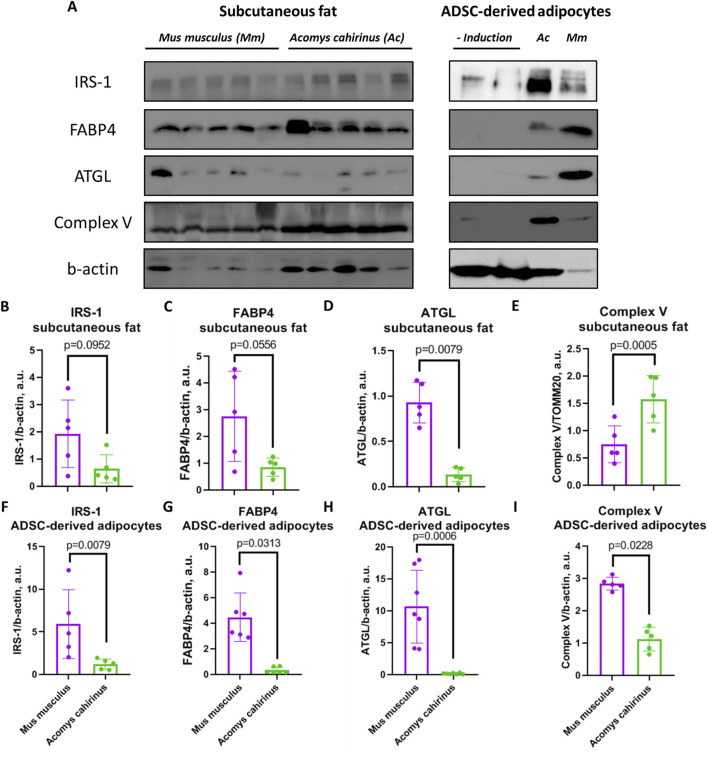
Comparison of metabolic protein expression in adipose tissue and adipocytes differentiated from ADSC of *Acomys cahirinus* and *Mus musculus.* **(A)** representative western blots of insulin receptor substrate 1 (IRS-1), fatty acid binding protein 4 (FABP4), adipocyte triglyceride lipase (ATGL) and ATP synthase F1 subunit alpha; (-Induction) - ADSC were cultured in standard medium without adipogenic inductors; **(B, F)** quantification of IRS-1 in adipose tissue and ADSC-derived adipocytes, respectively; **(C, G)** quantification of FABP4 in adipose tissue and ADSC-derived adipocytes, respectively; **(D, H)** quantification of ATGL in adipose tissue and ADSC-derived adipocytes, respectively; **(E, I)** quantification of ATP synthase F1 subunit alpha in adipose tissue and ADSC-derived adipocytes, respectively. Uncropped western blots with molecular weight markers are represented in Supplementary File 1. The data are presented as mean ± SD; Mann-Whitney rank sum U-test was used to calculate significance of differences; significance threshold was set at p < 0.05. All experiments have been performed with cells from 5 animals per group.

The expression of insulin receptor substrate 1 (IRS-1), which mediates insulin signaling, as well as fatty acid binding protein 4 (FABP4) was equal between *Acomys cahirinus* and *Mus musculus* in subcutaneous fat, but expression of two these proteins in ADSC-derived adipocytes was significantly lower in *Mus musculus* (Figures 7A–C,F,G). On the other hand, ATGL, which are responsible for lipolysis and fatty acid transport, was decreased in both fat samples and *in vitro* differentiated adipocytes of *Acomys cahirinus* (Figures 7A,D,H). In contrast, the alterations in the expression of ATP synthase F1 subunit alpha were inconclusive. An increase in its expression was observed in subcutaneous fat, while a decrease was noted in adipocyte cultures of *Acomys cahirinus* (Figures 7A,E,I).

## 4 Discussion

The present study investigates questions pertaining to the regenerative properties of ASDC and their involvement in adipose tissue self-renewal in *Acomys cahirinus*. The results obtained allow us to hypothesize that mature adipose tissue and ADSC-derived adipocytes of *Acomys cahirinus* display dissimilar properties. On the one hand, *Acomys cahirinus* ADSC demonstrate high proliferative activity and a shift in the adipo-osteogenesis balance, resulting in osteocyte formation; ADSC-derived adipocytes from *Acomys cahirinus* exhibit smaller LDs and a suppressed expression of adipogenic and bioenergetic adipocyte markers. In contrast, mature adipose tissue from *Acomys cahirinus* exhibits higher volume, low proliferation levels, enlarged adipocytes, and suppressed ATGL expression, yet enhanced expression of electron-transport chain (ETC) Complex V in comparison to *Mus musculus*.

First, *Acomys cahirinus* ADSC can be easily and effectively isolated from adipose tissue in accordance with established protocols involving mechanical disruption and enzymatic digestion. Isolated subcutaneous ADSC have a mesenchymal phenotype, as evidenced by the analysis of surface marker expression and the ability of ADSC to undergo three classic mesenchymal differentiations (Figure 1). This finding is consistent with previous studies of *Acomys cahirinus* ADSC (Dickinson et al., 2012). It is noteworthy that ADSC from different species exhibit species-specific characteristics in terms of cell surface marker expression. The present study demonstrates that *Acomys cahirinus* ADSC express CD73, CD90, and CD140b (PDGFRb). The expression of CD73 on ADSC has been observed in humans and small rodents, but not in large animals (Uder et al., 2018; Liu et al., 2016). CD90 expression has been detected in human and *Rattus norvegicus* ADSC, but not in *Mus musculus* ADSC (Periasamy et al., 2018). CD140b expression is present in human and small rodent ADSC (Doi et al., 2017; Baer et al., 2013; Muñoz et al., 2020). In conclusion, *Acomys cahirinus* ADSC exhibit a mesenchymal phenotype that is distinct from that of *Mus musculus* ADSC, and the expression of cell surface markers is more similar to *Rattus norvegicus* or humans.

As illustrated in Figure 2, the regenerative properties of *Acomys cahirinus* ADSC are markedly superior to those of *Mus musculus*. We found that enhanced *in vitro* wound healing in *Acomys cahirinus* is achieved through the activation of cell proliferation, but not through elevated migration (Figures 2D,E). The discrepancy in ADSC characteristics among species has already been documented and may be associated with variations in regeneration, longevity, and the incidence of disease between animals. For example, human ADSC have significantly higher survival in oxidative stress conditions (Cutler, 1991; Nahar et al., 2018). Proteomic data demonstrate enhanced expression of proteins involved into adhesion and locomotion biological processes in human ADSC in comparison to *Mus musculus* ADSC (Nahar et al., 2018). Therefore, human ADSC exhibit higher regenerative potential than small rodent ADSC. According to our results, *Acomys cahirinus* ADSC are more similar to human ADSC, which manifests as higher proliferation and wound healing in comparison to *Mus musculus* ADSC. A previous study confirmed this assertion by demonstrating that primary fibroblasts derived from *Acomys cahirinus* showed significantly higher proliferative activity than those derived from *Mus musculus* (Dill et al., 2023). Thus, in the context of proliferation and migration, *Acomys cahirinus* ADSC represents a better cell model for human-related research than *Mus musculus* ADSC.

The capacity of ADSCs to differentiate is a pivotal attribute for the processes of regeneration and self-renewal. Here, we investigate the balance between adipo- and osteogenesis in ADSC, because its role in human ADSC is closely related to metabolic homeostasis and the pathogenesis of type 2 diabetes (Skubis-Sikora et al., 2020; Agareva et al., 2022). In our study, *Acomys cahirinus* ADSC demonstrated suppressed adipogenesis and enhanced osteogenesis in comparison to *Mus musculus* ADSC (Figures 3, 4). Unfortunately, there is a paucity of information regarding adipogenesis in *Acomys cahirinus*. In line with our results, adipocyte formation in ears after injury has also been found to be downregulated and further impaired with age (Varholick et al., 2024). Such predominance of osteogenic differentiation in human ADSC is known to impair adipogenesis and energy deposition in fat depots leading to metabolic abnormalities and hyperglycemia. Despite this, blood glucose level in *Acomys cahirinus* after a short starvation period is significantly lower than in *Mus musculus* and tends to be decreased after longer fasting (Figure 5D)*.* It can therefore be proposed that the shift in differentiation balance toward osteogenesis is not involved in the maintenance of metabolic homeostasis in *Acomys cahirinus*.

Despite a reduction in ADSC adipogenesis, the relative adipose tissue content (normalized on body weight) is significantly higher in *Acomys cahirinus* in comparison to *Mus musculus*, with a similar Lee index, which is an analog of BMI in rodents (Figures 5A–C). Recent study has also reported excessive adipose tissue deposition in *Acomys cahirinus* skin (Jiang et al., 2019). These data raise the question regarding the mechanism of elevated adipose tissue growth in combination with impaired adipogenesis in *Acomys cahirinus*. To address this phenomenon, we analyzed the intact subcutaneous adipose tissue of the *Acomys cahirinus*. Our findings indicate that the number of proliferating cells in *Acomys cahirinus* adipose tissue is reduced in comparison to *Mus musculus* (Figure 6A). Moreover, *Acomys cahirinus* adipocytes demonstrated large size in comparison to *Mus musculus* (Figure 6B). It appears that the elevated adipose tissue content observed in *Acomys cahirinus* is attributable to an increase in the size and lipid storage in existing adipocytes rather than the formation of new cells. Taken together, *Acomys cahirinus* exhibits adipocytes enlargement and a shift in the adipo-osteogenesis balance towards osteogenesis, which are characteristics associated with glucose metabolism abnormalities in humans. Nevertheless, the blood glucose levels of *Acomys cahirinus* are found to be lower in comparison to *Mus musculus*. This raises the question of which molecular mechanisms enable *Acomys cahirinus* to maintain normal glucose tolerance in conjunction with large adipocyte’s size and impaired adipogenesis.

We suggest that unique metabolic properties of *Acomys cahirinus* mature adipocytes may be involved in their ability to maintain normoglycemia during obesity. In order to gain an insight into regulation of metabolism in *Acomys cahirinus* adipocytes, we have analyzed the expression of biochemical markers related to insulin signaling (IRS-1), lipid metabolism (FABP4 and ATGL) and mitochondrial ETC (Complex V). IRS-1 is a crucial mediator of insulin signaling, which controls glucose uptake and lipid synthesis (Wang et al., 2009). IRS-1 was significantly lower in *Acomys cahirinus* ADSC-derived adipocytes and non-significantly lower in case of adipose tissue biopsies (Figures 7A,B,F). This may indicate that *Acomys cahirinus* ADSC-derived adipocytes can have lower activity of insulin signaling and insulin-dependent glucose uptake than *Mus musculus* ADSC-derived adipocytes. However, expression of signaling components may not influence signaling activity. FABP4 is a protein that plays a role in the influx of fatty acids into cells (Garin-Shkolnik et al., 2014). The expression of FABP4 was found to be significantly lower in *Acomys cahirinus* ADSC-derived adipocytes and non-significantly lower in mature adipose tissue (Figures 7A,C,G). Thus, fatty acids transport in *Acomys cahirinus* ADSC-derived adipocytes is impaired in comparison to *Mus musculus*. Expression of ATGL, a crucial lipolytic enzyme, was significantly decreased in both *Acomys cahirinus* ADSC-derived adipocytes and mature adipose tissue (Figures 7A,D,H). ATGL inhibition can play a protective role, preventing excessive constitutive lipolysis and consequent development of non-alcoholic fatty liver disease (Schweiger et al., 2017). In case of *Acomys cahirinus* large adipocytes, protection against enhanced lipolysis can be a crucial species-specific mechanism maintaining systemic energy homeostasis. The only discrepancies between the ADSC-derived adipocytes and adipose tissue were observed in the expression of ATP-synthase and Complex V (Figures 7A,E,I). The expression of ATP-synthase represents the efficiency of ATP synthesis in cells and may reflect their energy demand (Arakaki et al., 2007). *Acomys cahirinus* ADSC-derived adipocytes have suppressed ATP-synthase expression, whereas *Acomys cahirinus* mature adipose tissue has enhanced ATP-synthase level in comparison to respective cells from *Mus musculus*. This contradiction can be explained by the presence of a heterogeneous cellular composition within whole mount adipose tissue, which can impact the results. It is our contention that the most intriguing molecular characteristic of *Acomys cahirinus* ADSC-derived adipocytes and mature adipose tissue is the diminished expression of ATGL, which may serve as a defense mechanism against the ectopic fat accumulation during obesity.

In summary, the most insightful result of the present study is the formulation of a hypothesis regarding the protective mechanisms that enable *Acomys cahirinus* to maintain metabolic homeostasis despite impaired adipogenesis and large adipocytes. *Acomys cahirinus* ADSC have high regenerative potential together with predisposition to osteogenesis, but not to adipogenesis, which can be a reason of adipocyte’s enlargement. *Acomys cahirinus* has protective mechanisms that can be related to suppressed ATGL expression, altered immune response, enhanced metabolic energy wasting cycles, and T3 and T4 synthesis (Shafrir, 2000; Made et al., 2020; Shafrir and Adler, 1983; Mamrot et al., 2017). Further studies should be conducted to analyze the enumerated aspects of *Acomys cahirinus* metabolism and insulin-dependent tissues physiology.

## 5 Conclusion

In the present study we highlight that the existence of species-specific mechanisms of maintaining metabolic homeostasis during obesity depends on adipose tissue plasticity. We have compared regenerative properties of classic small lab animal model *Mus musculus* and *Acomys cahirinus*, small animal with unique regeneration. We suggest *Acomys cahirinus* as a promising model for studying protective mechanisms against glucose intolerance, as this rodent exhibits normoglycemia despite elevated adiposity. Surprisingly, we found that despite enhanced regeneration and ADSC proliferation *Acomys cahirinus* exhibit adipocyte’s enlarged size, lower level of adipogenesis and activated osteogenesis in comparison to *Mus musculus*. We propose that exceptional adipocytes metabolic properties of *Acomys cahirinus*, including decreased ATGL expression, may serve as a protective mechanism against obesity associated metabolic complications. Further investigation of these phenomena may facilitate the elucidation of the underlying mechanisms, which can then be employed in the development of novel therapeutic approaches.

## Data Availability

The raw data supporting the conclusions of this article will be made available by the authors, without undue reservation.

## Abbreviations

ADSC, adipose-derived stem cells; ATGL, adipose tissue triglyceride lipase; ATP, adenosine triphosphate; BMI, body mass index; DMEM LG, Dulbecco’s modified Eagle medium low glucose; DAPI, 4′,6-diamidino-2-phenylindole; ETC, electron transport chain; FBS, fetal bovine serum; FOV, field of view; FABP4, fatty acids binding protein type 4; IBMX, isobutylmethylxanthine; IRS-1, insulin receptor substrate; LDs, lipid droplets; MTT, 3-(4,5-dimethylthiazol-2-yl)-2,5-diphenyltetrazolium bromide; PCNA, proliferating cell nuclear antigen; PVDF, polyvinylidene difluoride; PFA, paraformaldehyde; PDGFRb, platelet-derived growth factor receptor beta; RT PCR, real time polymerase chain reaction; RIPA, radioimmunoprecipitation assay buffer; SDS-PAGE, sodium dodecylsulfate polyacrylamide gel electrophoresis; SEM, standard error mean; TAG, triacylglyceride.
