# Supplementary material for: Osteogenic shift in the adipose-derived stem cells of Acomys cahirinus is linked to impaired adipose tissue self-renewal
Source: Front Cell Dev Biol. 2025 Jul 30;13:1603405. doi: 10.3389/fcell.2025.1603405 (PMC12343677; doi:10.3389/fcell.2025.1603405)
Supplement: Supplementary file 1 [file Table1.docx]

Table 1. Sequences of primers for RT PCR

|  | Mus musculus | Acomys cahirinus |
| --- | --- | --- |
| RUNX2 | | |
| Forward primer | TCGCCTCACAAACAACCCAG | AGGTAGGTGTGGTAGTGAGTG |
| Reverse primer | TGCTTGCAGCCTTAAATATTCCTG | TCTCAGTAAGAAGAGCCAGGCA |
| Col1a1 | | |
| Forward primer | CCAGCCGCAAAGAGTCTACAT | ATGGGAGGAGAGAATGCCAAC |
| Reverse primer | ATACCTCGGGTTTCCACGTC | GAGAGGCTGAGGGAGGGTTT |
| SPP1 | | |
| Forward primer | CTGGCTGAATTCTGAGGGACT | TTCTGAGATGGGTCCGGCTT |
| Reverse primer | TTCTGTGGCGCAAGGAGATT | TCAGCATCCTTGCAGCTTTG |
| AdipoQ | | |
| Forward primer | CTTGGTCCTAAGGGTGAGACAG | TCCCTGGTCTCCACGATGTT |
| Reverse primer | AGTGCTGCCGTCATAATGATTC | ACTCCAGACACTCACAAGCC |
| ATGL | | |
| Forward primer | CACCGTCCAAGACAGGTCAAGTAGA | GTCCCGCACTAAAACACCAC |
| Reverse primer | AAACTCTACTTGACCTGTCTTGGAC | GCCACGCCAATGTGGTAGA |
| PPARg | | |
| Forward primer | TCTCAGAGGGCCAAGGATTC | GGAGCAGAGATGCTGGAGAAA |
| Reverse primer | GCAGCAGGTTGTCTTGGATG | GCATCAAATCCTGAGCCTTGAAC |
| Beta-actin | | |
| Forward primer | AGTGTGACGTTGACATCCGTA | TCGTTCACCGCAAATGCTTC |
| Reverse primer | GCCAGAGCAGTAATCTCCTTCT | GCCTTCACCGTTCCAGTTTTT |
