# Supplementary material for: Osteogenic shift in the adipose-derived stem cells of Acomys cahirinus is linked to impaired adipose tissue self-renewal
Source: Front Cell Dev Biol. 2025 Jul 30;13:1603405. doi: 10.3389/fcell.2025.1603405 (PMC12343677; doi:10.3389/fcell.2025.1603405)

Blotting Acomys, C57BL/6J

(Subcutaneous fat, white adipogenesis)

OXPHOS (complex V), IRS-1, FABP4, ATGL

Ac, -I = Acomys, without Induction
Bl, -I = C57BL/6J, without induction
Ac, WI = Acomys, white adipogenesis
Bl, WI = C57BL/6J, white adipogenesis

Subcutaneous fat
OXPHOS (complex V), repeat 1

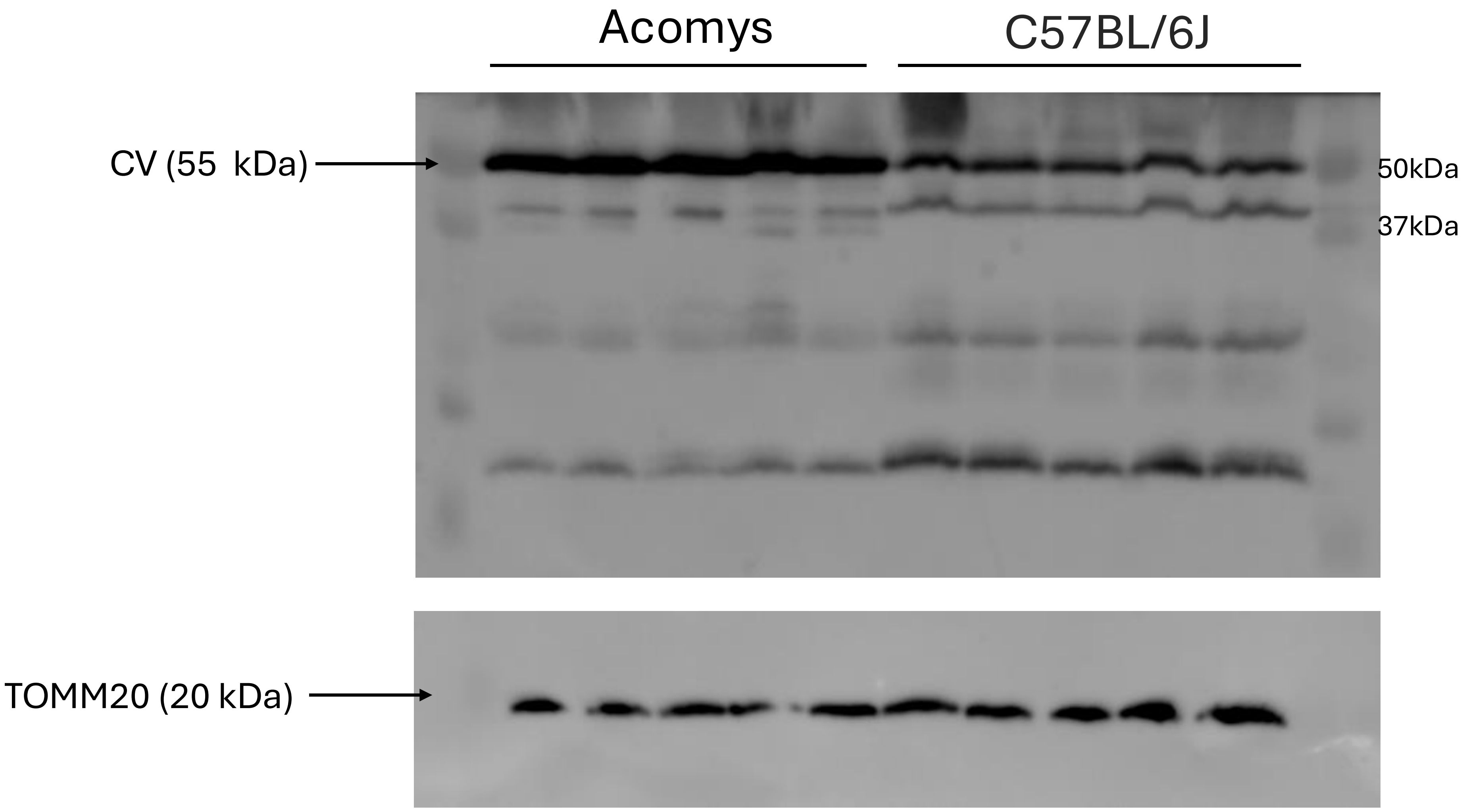

Subcutaneous fat
OXPHOS (complex V), repeat 2

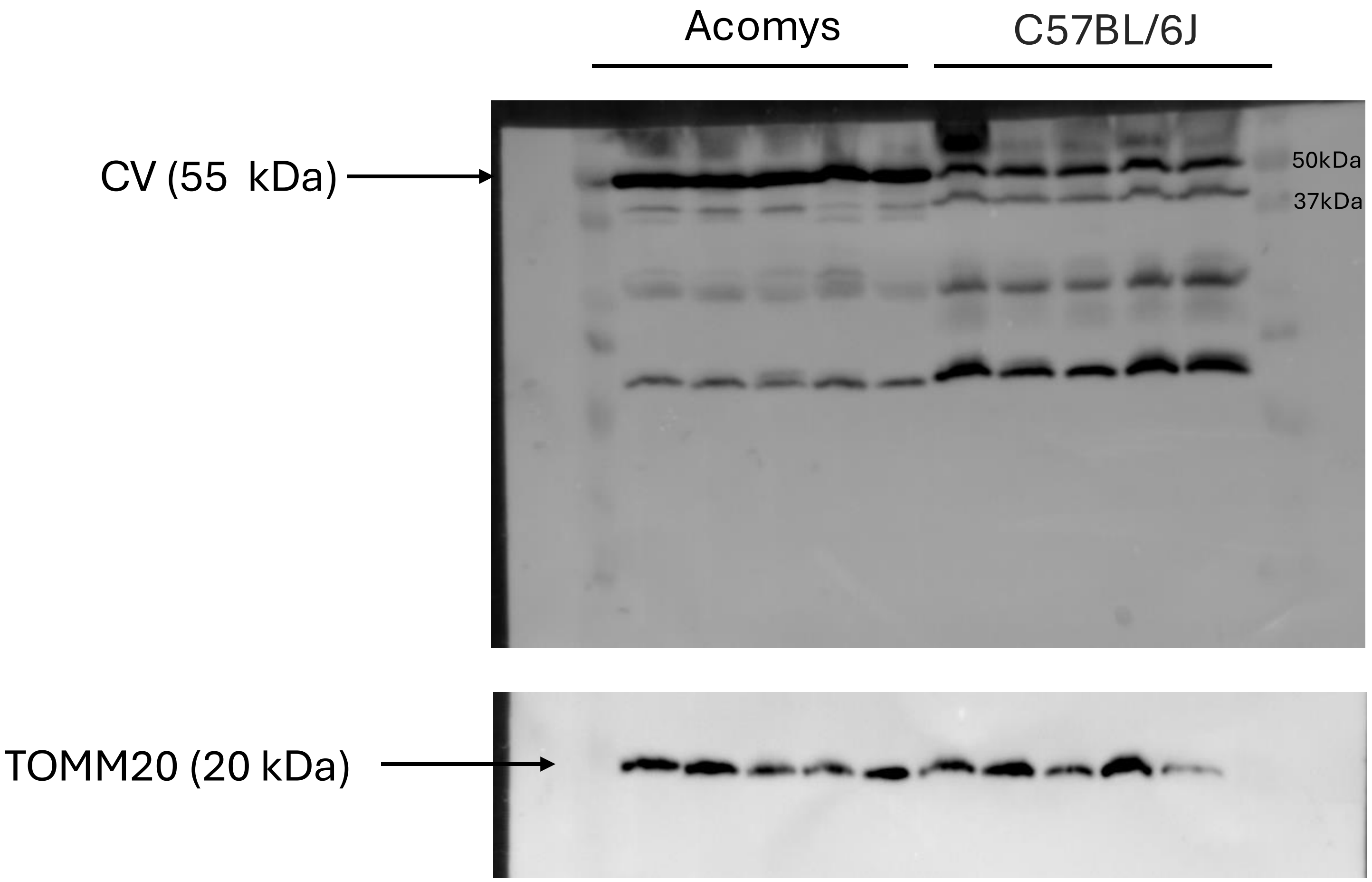

Subcutaneous fat
IRS-1, repeat 1

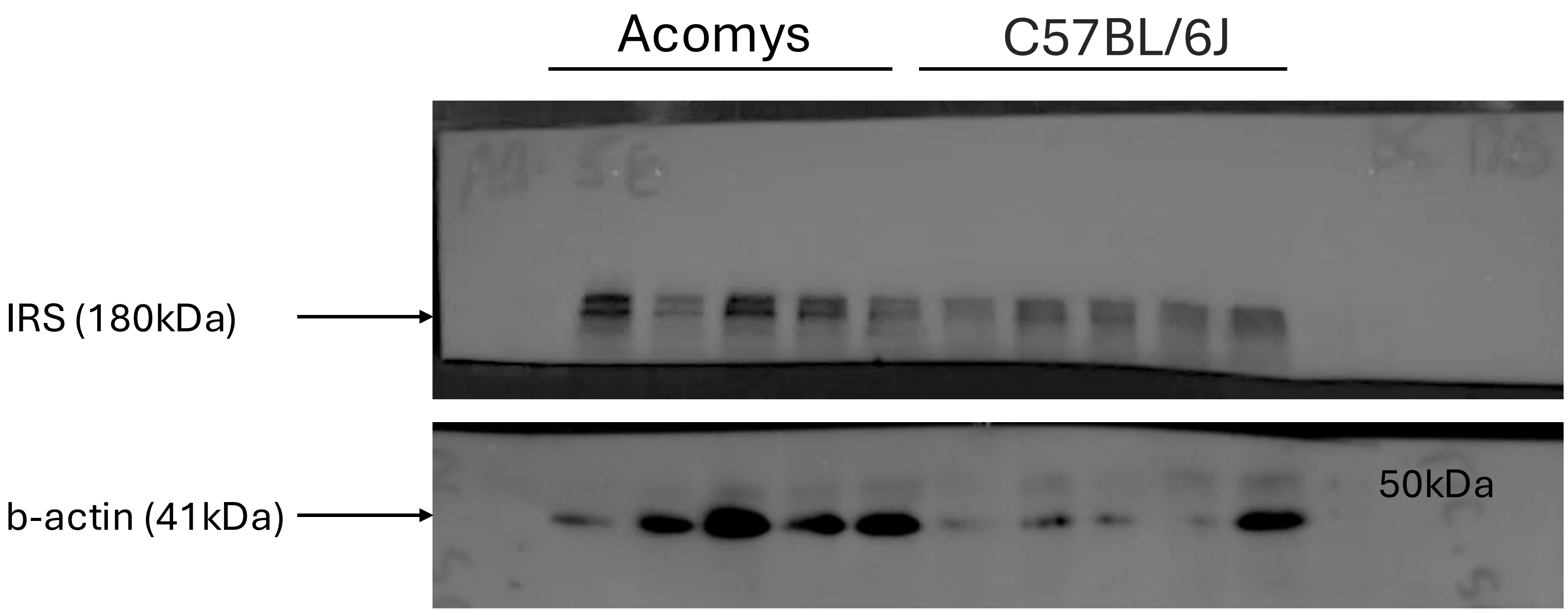

Subcutaneous fat
IRS-1, repeat 2

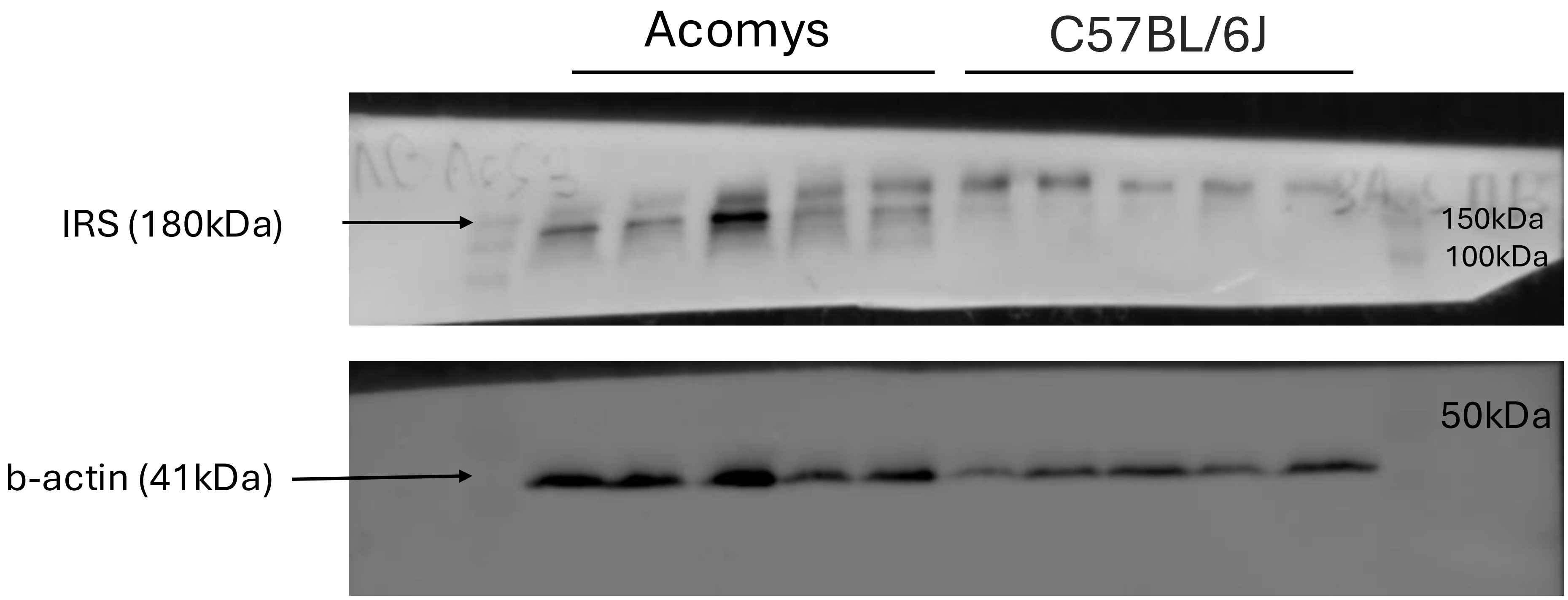

Subcutaneous fat
FABP4, repeat 1

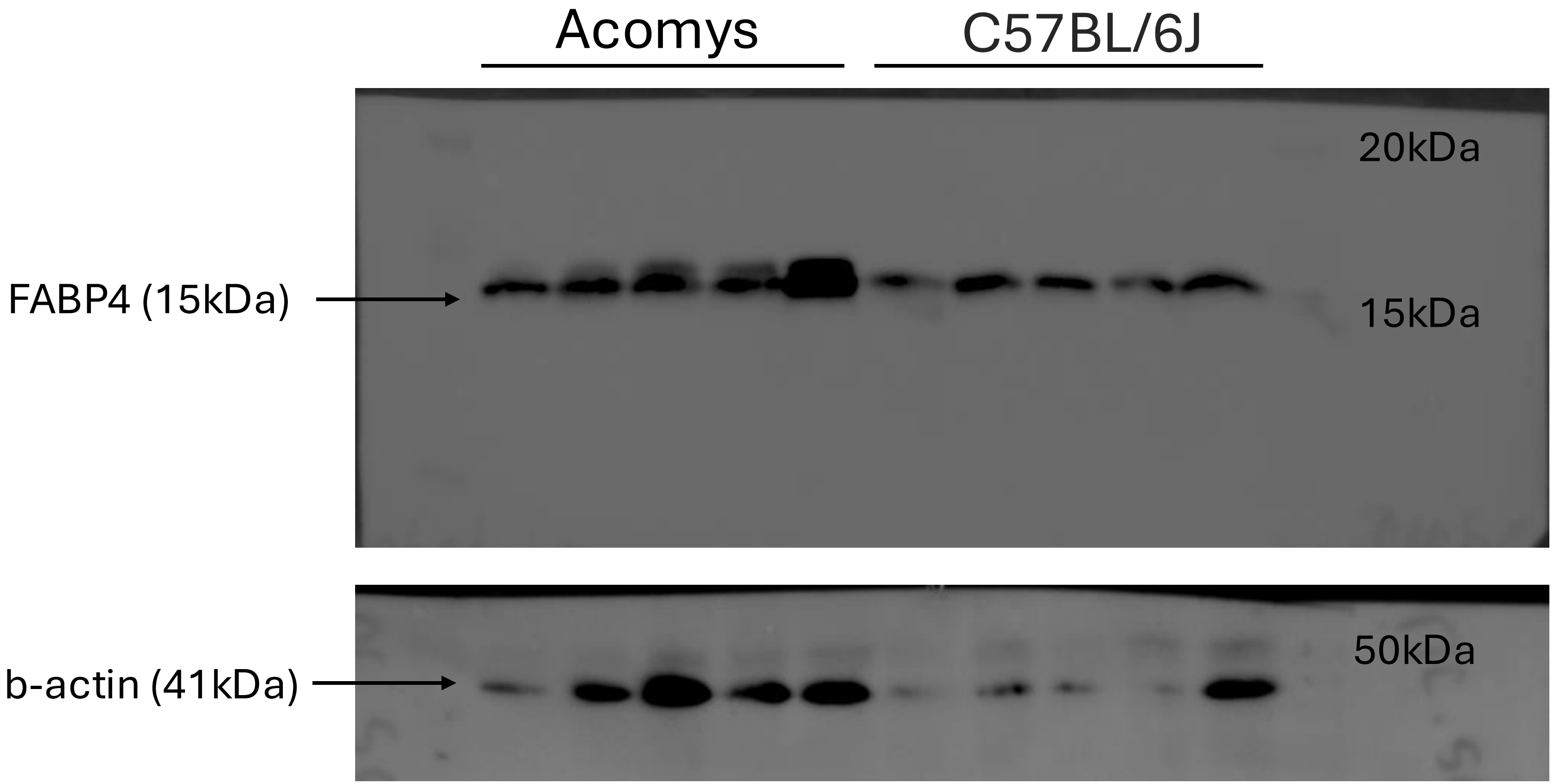

Subcutaneous fat
FABP4, repeat 2

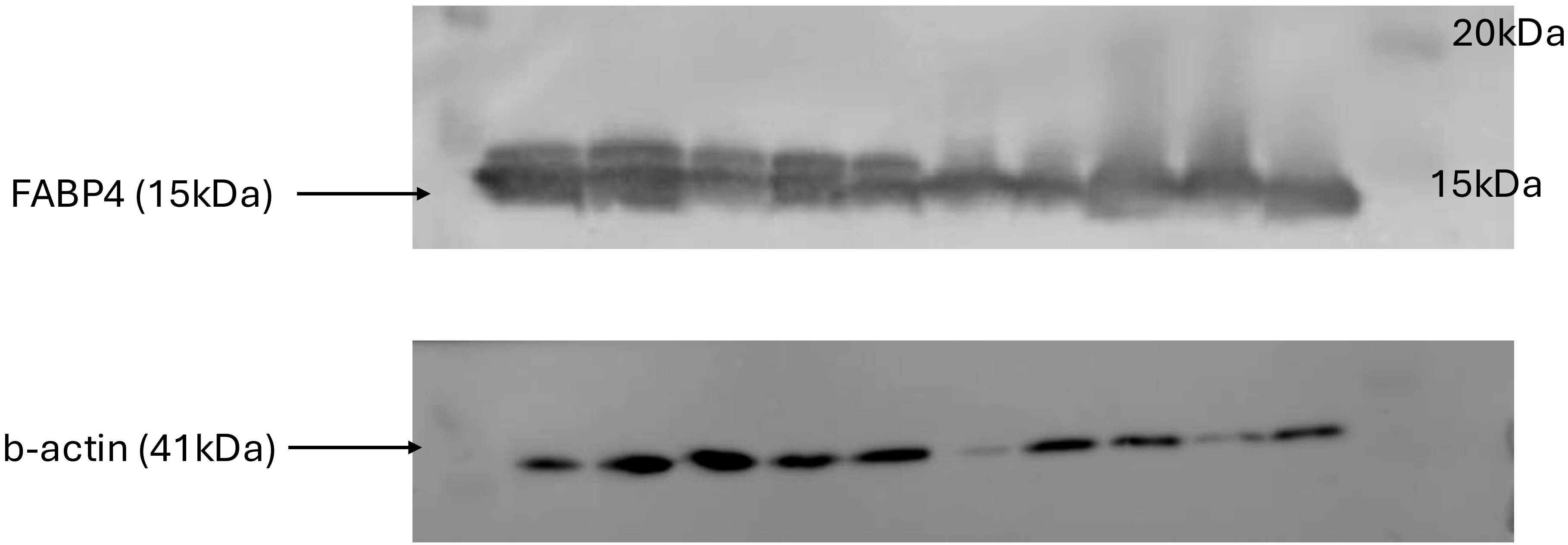

Subcutaneous fat
ATGL, repeat 1

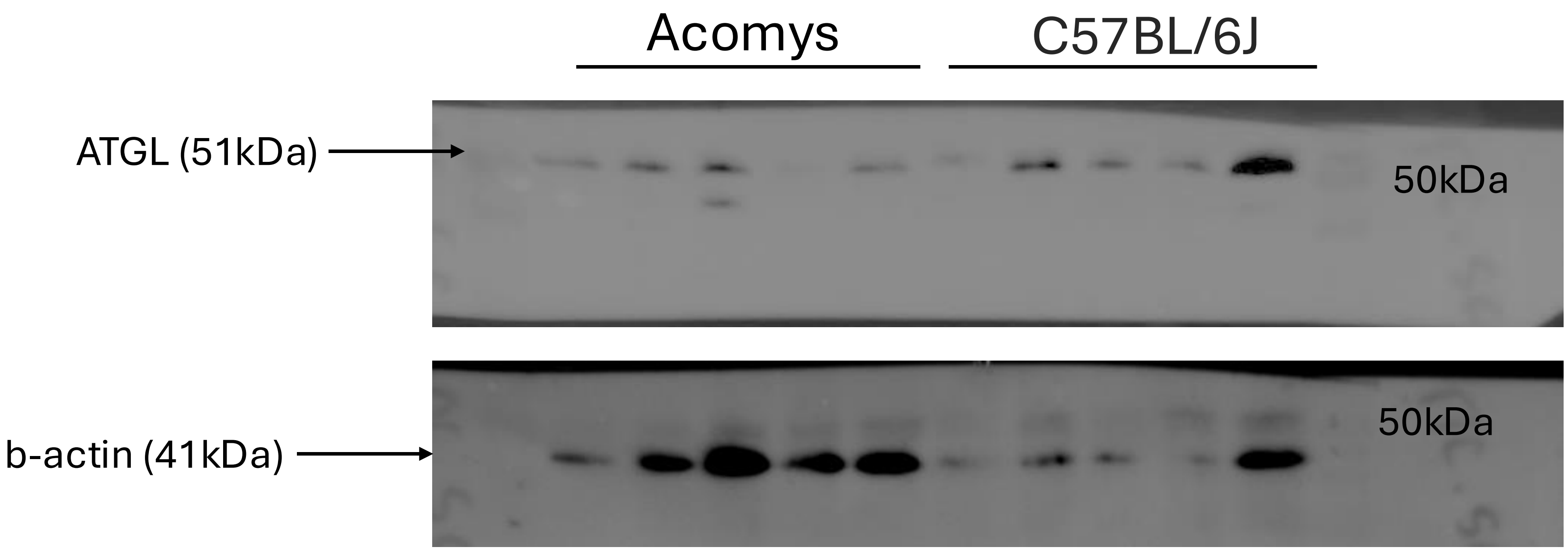

Subcutaneous fat
ATGL, repeat 2

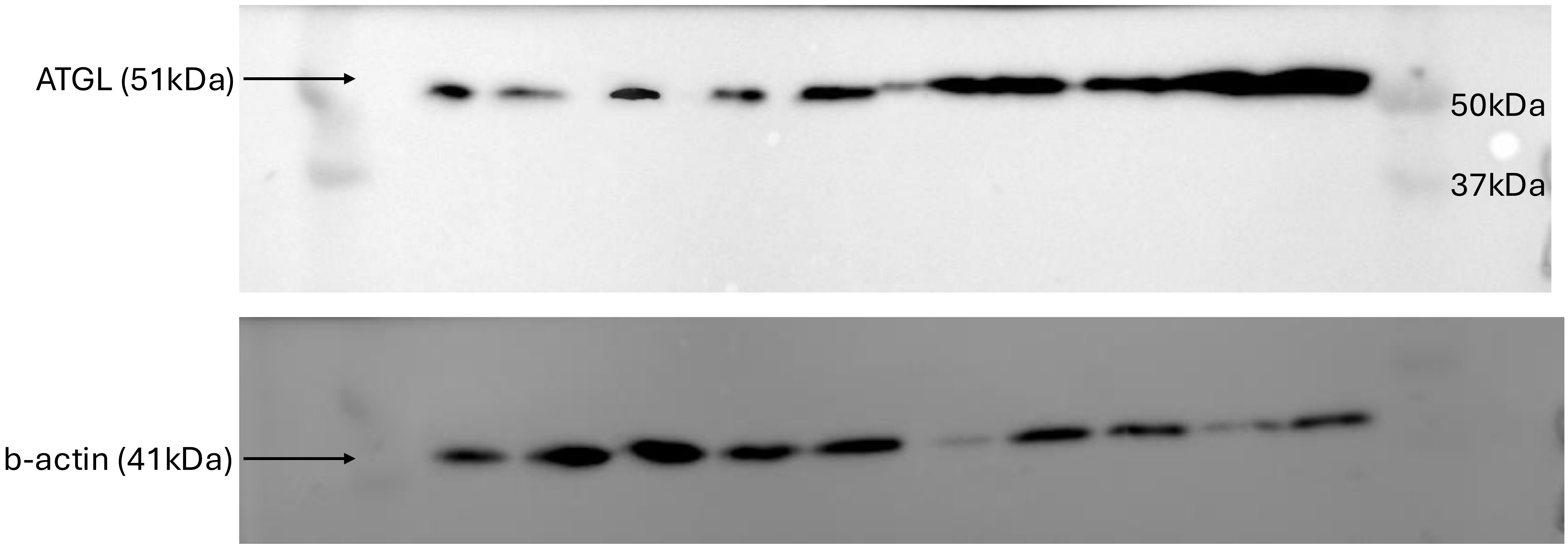

White adipogenesis
OXPHOS (complex V), repeat 1

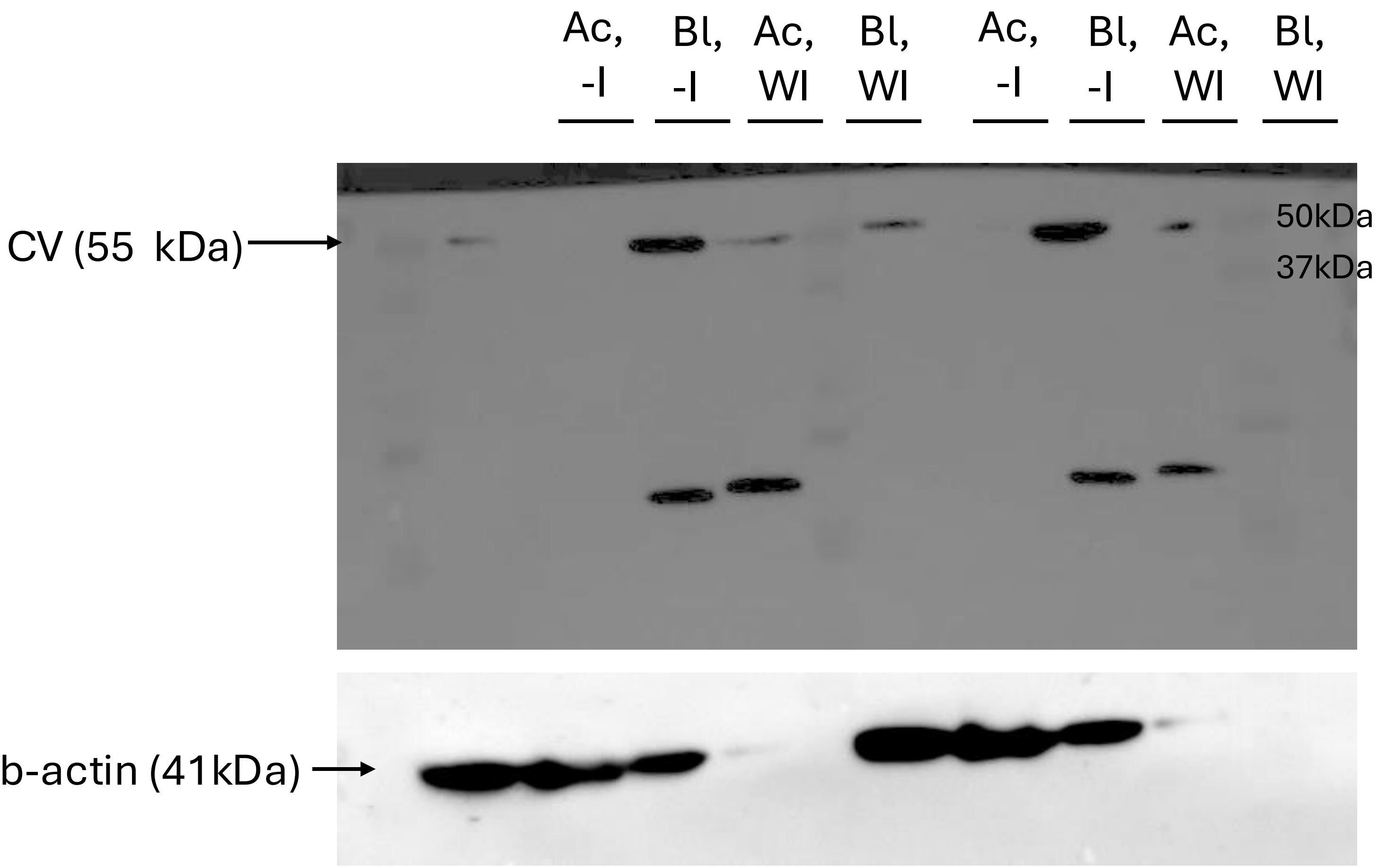

White adipogenesis
OXPHOS (complex V), repeat 2

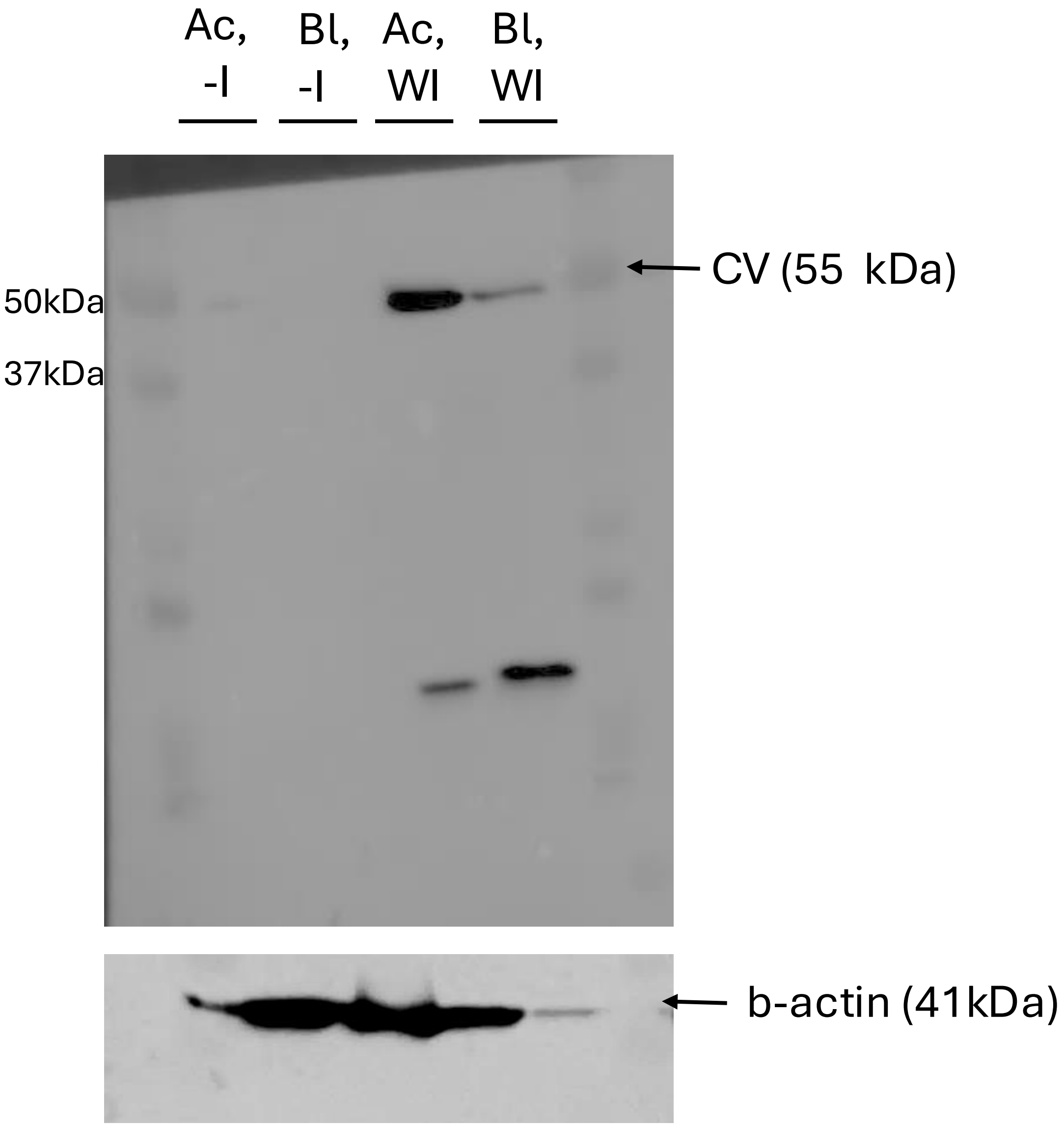

White adipogenesis
IRS-1

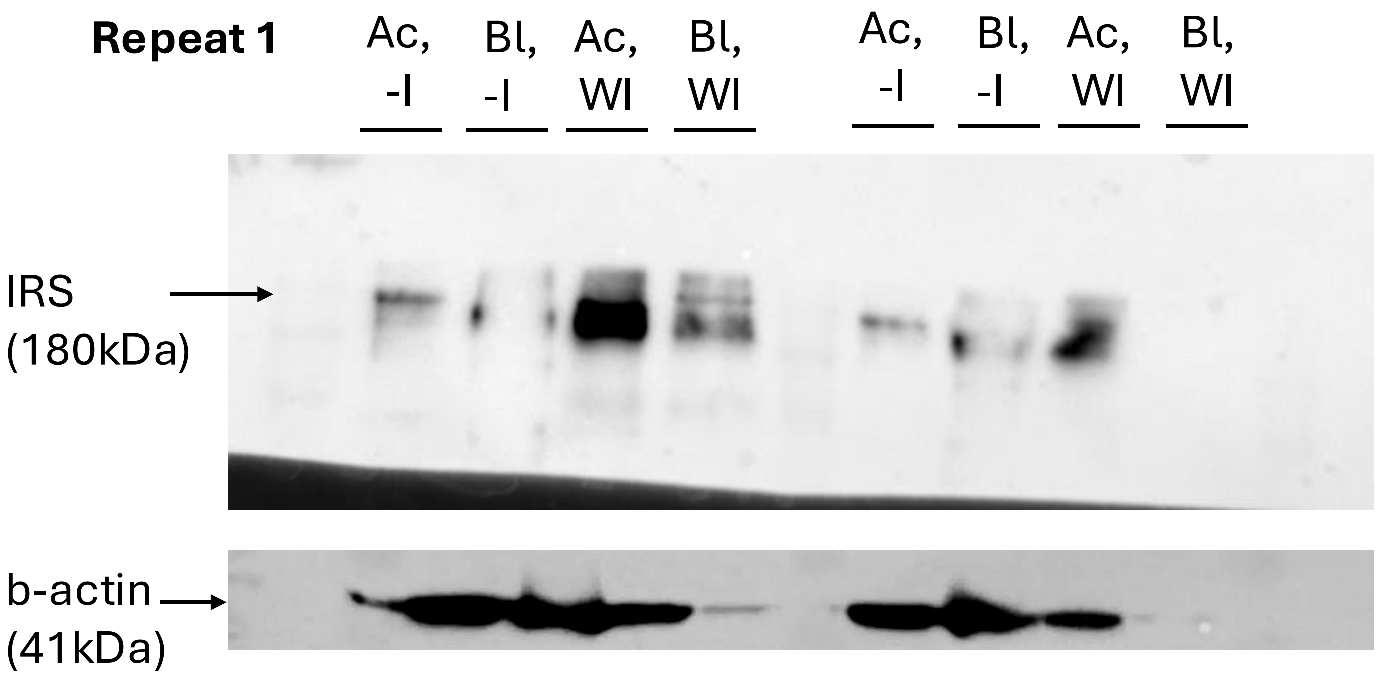

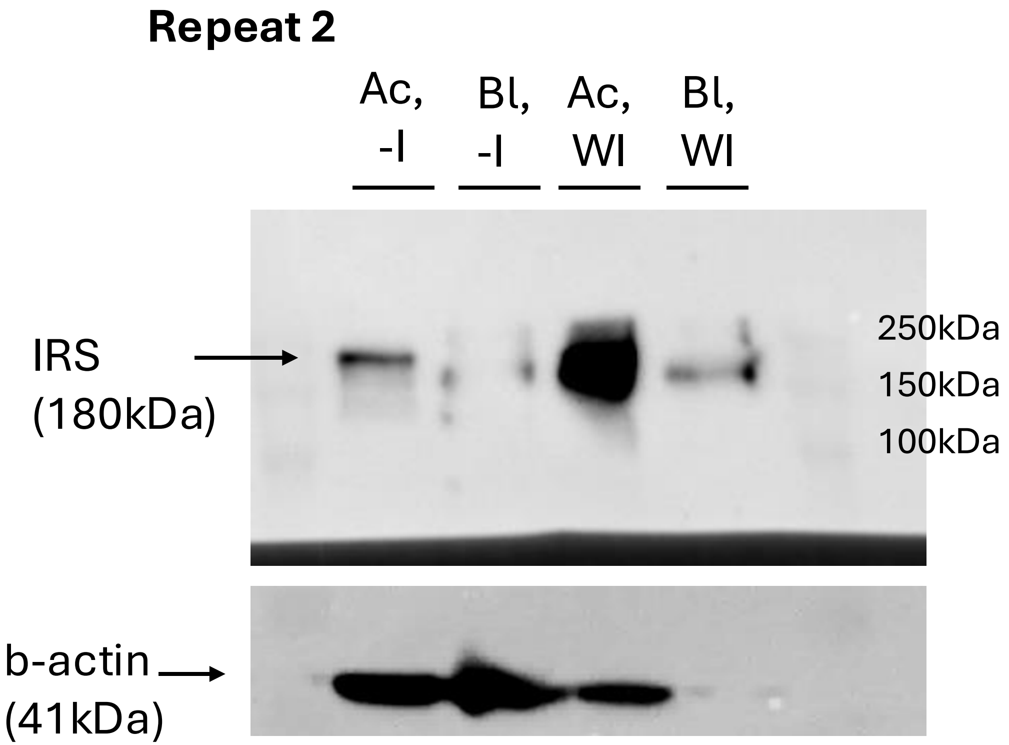

White adipogenesis
FABP4, repeat 1

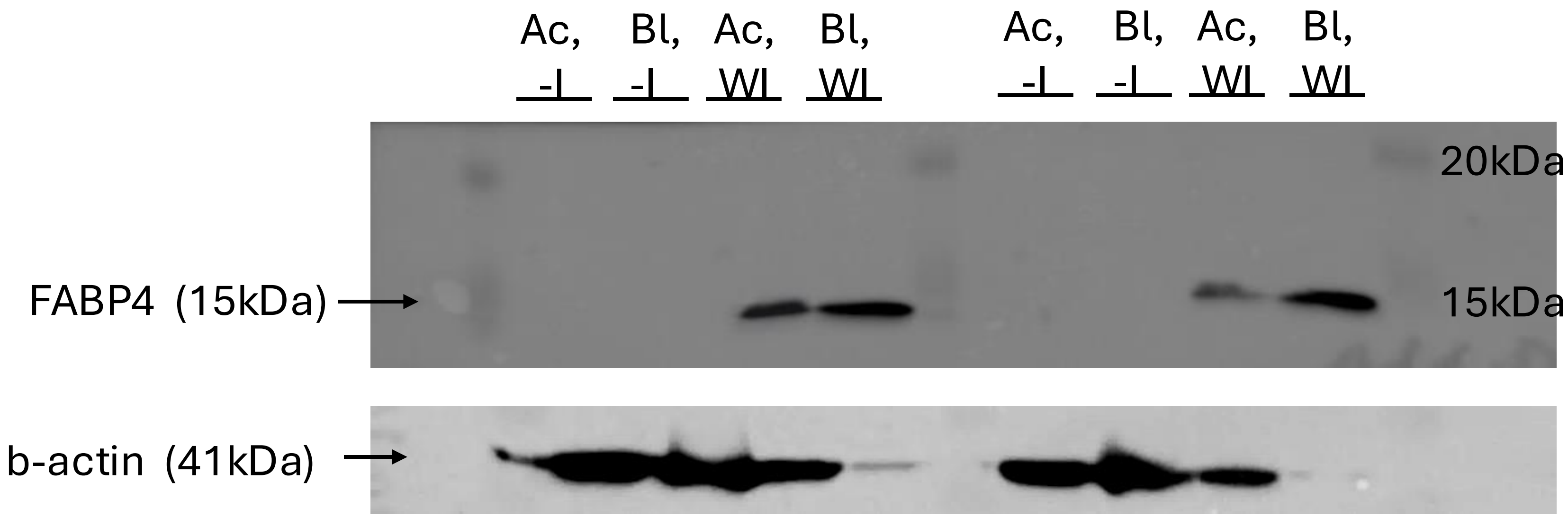

White adipogenesis
FABP4, repeat 2

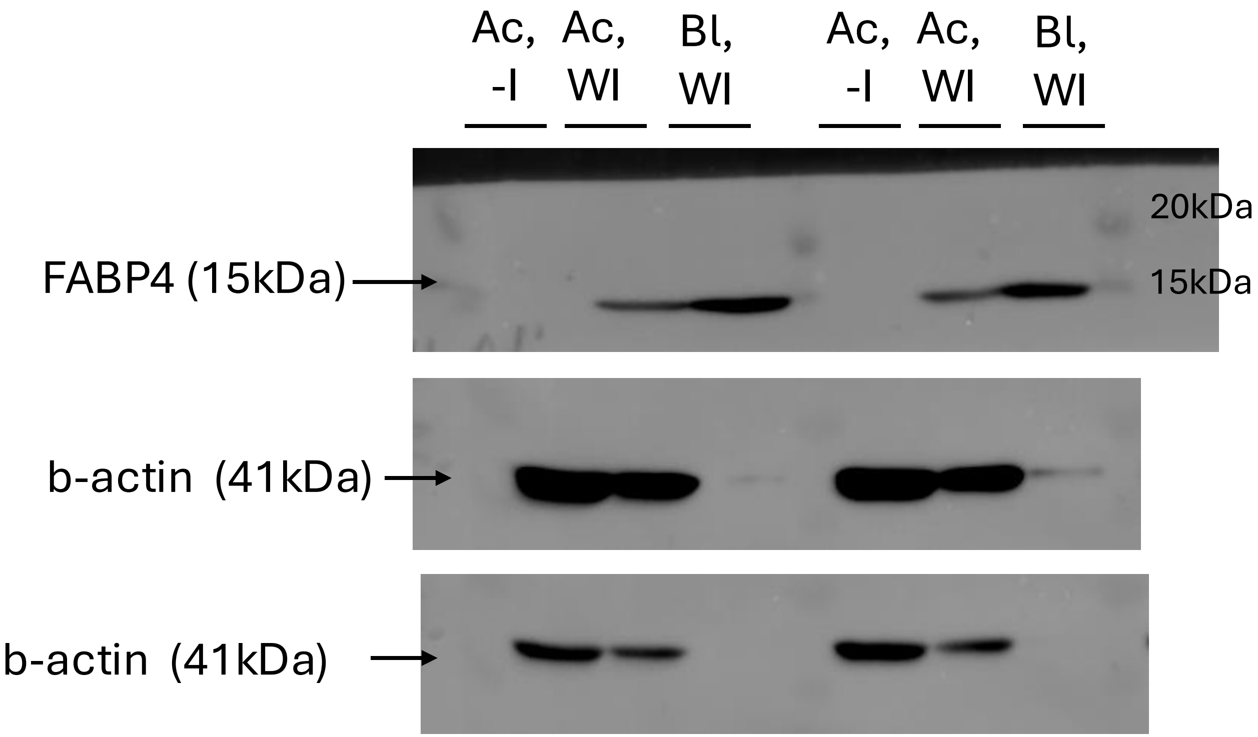

White adipogenesis
ATGL, repeat 1

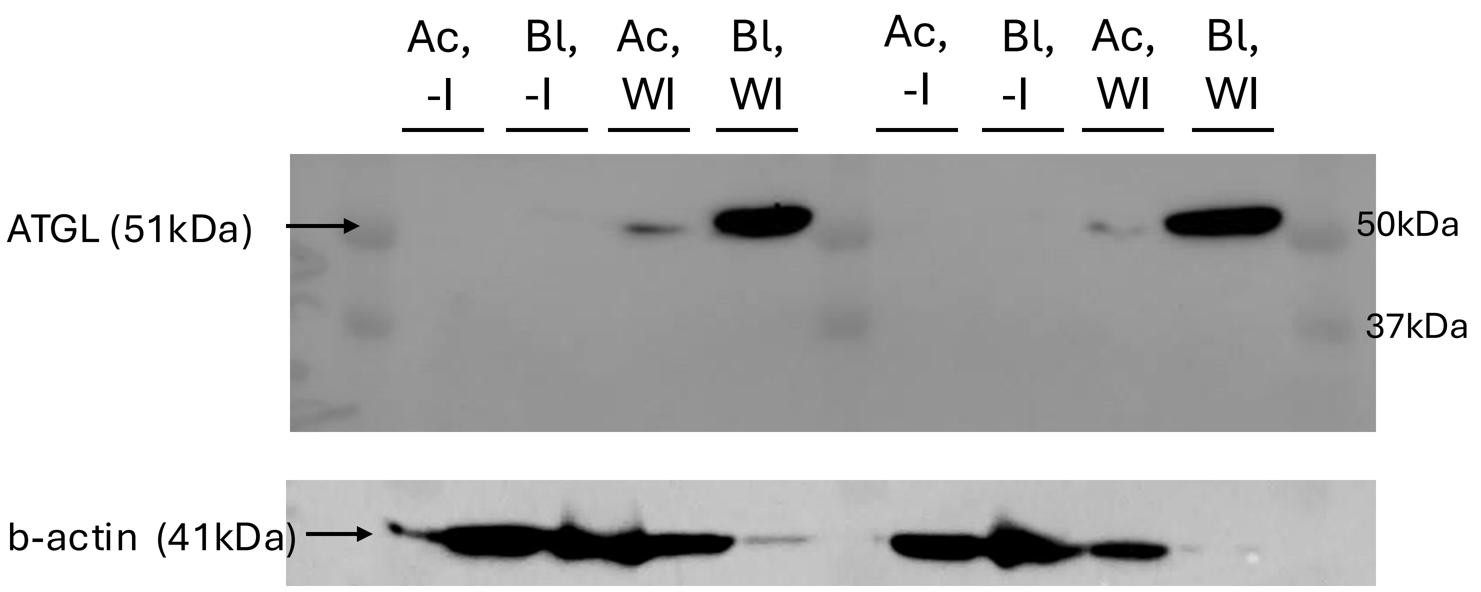

White adipogenesis
ATGL, repeat 2

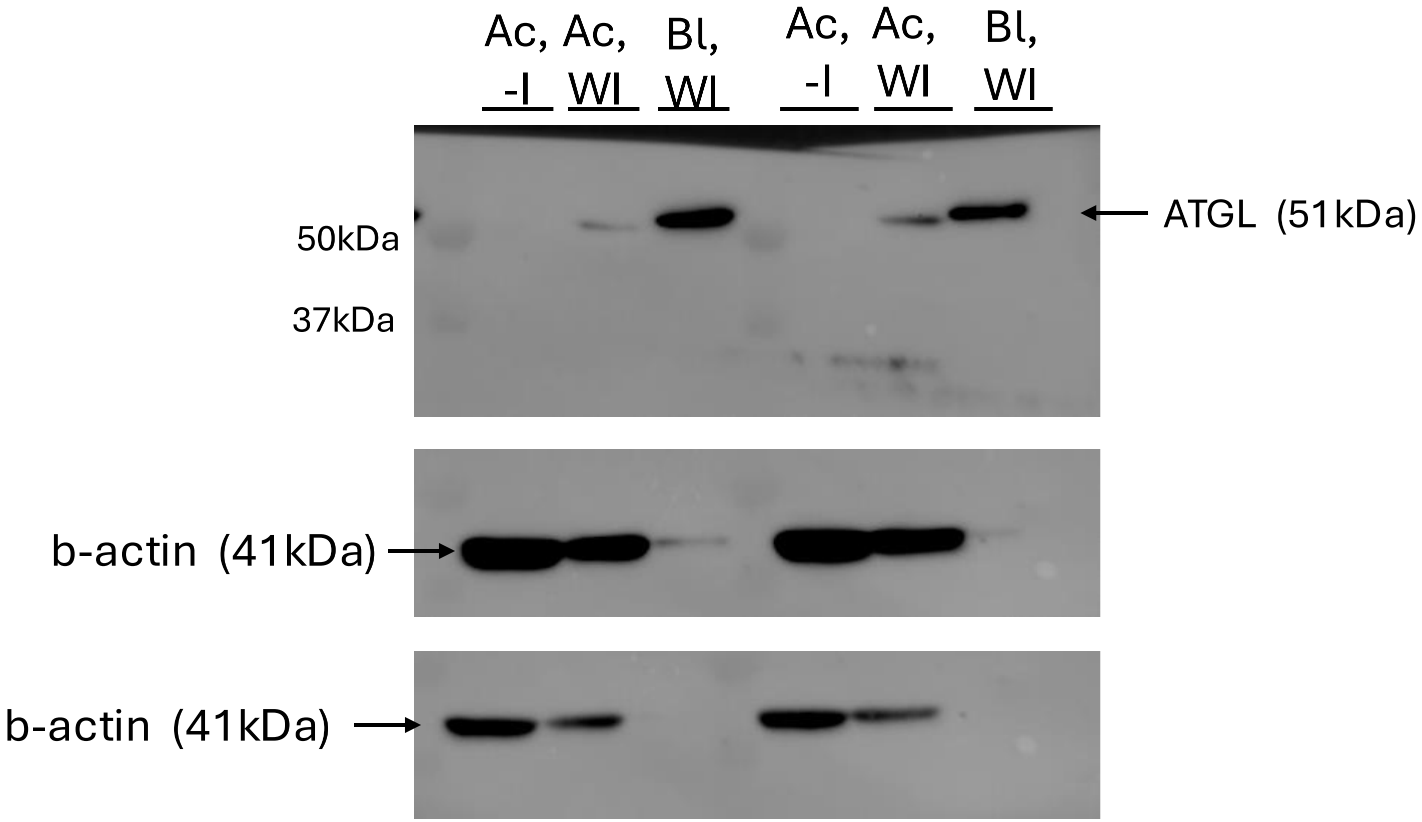

Supplement: Supplementary file 2 [file DataSheet1.pdf]
